# HER2 heterogeneity and treatment response–associated profiles in HER2-positive breast cancer in the NCT02326974 clinical trial

**DOI:** 10.1172/JCI176454

**Published:** 2024-02-01

**Authors:** Zheqi Li, Otto Metzger Filho, Giuseppe Viale, Patrizia dell’Orto, Leila Russo, Marie-Anne Goyette, Avni Kamat, Denise A. Yardley, Vandana Gupta Abramson, Carlos L. Arteaga, Laura M. Spring, Kami Chiotti, Carol Halsey, Adrienne G. Waks, Tari A. King, Susan C. Lester, Jennifer R. Bellon, Eric P. Winer, Paul T. Spellman, Ian E. Krop, Kornelia Polyak

**Affiliations:** 1 Department of Medical Oncology, Dana-Farber Cancer Institute, Boston, Massachusetts, USA.; 2 Department of Medicine, Brigham and Women’s Hospital, Boston, Massachusetts, USA.; 3 Department of Medicine, Harvard Medical School, Boston, Massachusetts, USA.; 4 Division of Pathology, European Institute of Oncology, IRCCS, Milan, Italy.; 5 University of Milan, School of Medicine, Milan, Italy.; 6 Harvard University, Cambridge, Massachusetts, USA.; 7 Sarah Cannon Research Institute and Tennessee Oncology, Nashville, Tennessee, USA.; 8 Department of Medicine, Vanderbilt–Ingram Cancer Center, Nashville, Tennessee, USA.; 9 University of Texas Southwestern, Simmons Comprehensive Cancer Center, Dallas, Texas, USA.; 10 Department of Medical Oncology, Massachusetts General Hospital, Boston, Massachusetts, USA.; 11 Department of Molecular and Medical Genetics, Oregon Health and Science University, Portland, Oregon, USA.; 12 Department of Pathology, Brigham and Women’s Hospital, Boston, Massachusetts, USA.; 13 Harvard Medical School, Boston, Massachusetts, USA.; 14 Department of Internal Medicine, Yale Cancer Center, New Haven, Connecticut, USA.

**Keywords:** Oncology, Breast cancer

## Abstract

**BACKGROUND:**

HER2-targeting therapies have great efficacy in HER2-positive breast cancer, but resistance, in part due to HER2 heterogeneity (HET), is a significant clinical challenge. We previously described that in a phase II neoadjuvant trastuzumab emtansine (T-DM1) and pertuzumab (P) clinical trial in early-stage HER2-positive breast cancer, none of the patients with HER2-HET tumors had pathologic complete response (pCR).

**METHODS:**

To investigate cellular and molecular differences among tumors according to HER2 heterogeneity and pCR, we performed RNA sequencing and *ERBB2* FISH of 285 pretreatment and posttreatment tumors from 129 patients in this T-DM1+P neoadjuvant trial. A subset of cases was also subject to NanoString spatial digital profiling.

**RESULTS:**

Pretreatment tumors from patients with pCR had the highest level of *ERBB2* mRNA and ERBB signaling. HER2 heterogeneity was associated with no pCR, basal-like features, and low *ERBB2* expression yet high ERBB signaling sustained by activation of downstream pathway components. Residual tumors showed decreased HER2 protein levels and *ERBB2* copy number heterogeneity and increased PI3K pathway enrichment and luminal features. HET tumors showed minimal treatment-induced transcriptomic changes compared with non-HET tumors. Immune infiltration correlated with pCR and HER2-HET status.

**CONCLUSION:**

Resistance mechanisms in HET and non-HET tumors are distinct. HER2-targeting antibodies have limited efficacy in HET tumors. Our results support the stratification of patients based on HET status and the use of agents that target downstream components of the ERBB signaling pathway in patients with HET tumors.

**TRIAL REGISTRATION:**

ClinicalTrials.gov NCT02326974.

**FUNDING:**

This study was funded by Roche and the National Cancer Institute.

## Abstract 2

HER2 heterogeneous and non-HER2 heterogeneous tumors show distinct resistance mechanisms and the immune environment influences the response to neoadjuvant T-DM1 plus pertuzumab treatment.

## Graphical abstract

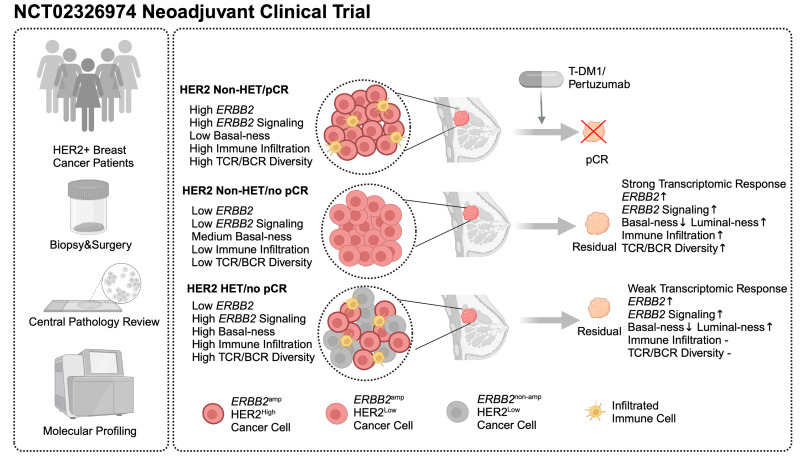

## Introduction

Breast cancer is one of the most prevalent and life-threatening malignancies in women worldwide (1). Breast cancer is a heterogeneous disease clinically classified on the basis of the expression of estrogen and progesterone hormone receptors (ER and PR) and human epidermal growth factor receptor 2 (HER2; encoded by *ERBB2*), which guides treatment. HER2-targeted therapies including monoclonal antibodies and antibody-drug conjugates (ADCs) used alone and in combination with chemotherapy have been highly effective for the treatment of HER2-positive tumors (2). T-DM1 is a conjugate of trastuzumab, a monoclonal antibody targeting HER2, and the potent cytotoxic agent emtansine, allowing for targeted delivery of the cytotoxic payload directly to HER2-positive cells, sparing normal tissues and reducing off-target toxicities (3). Pertuzumab is a HER2-targeted monoclonal antibody that is used clinically in combination with trastuzumab-based therapies.

Despite the remarkable success of HER2-targeting ADCs, resistance to treatment remains a clinical challenge requiring the design of combination therapies. We and others have previously conducted clinical trials using T-DM1 and pertuzumab, including the neoadjuvant study discussed herein (NCT02326974), which have demonstrated that a proportion of patients do not respond or eventually develop resistance to combined T-DM1 and pertuzumab treatment, leading to disease progression (4–6). We previously described that HER2 heterogeneity (defined by American Society of Clinical Oncology [ASCO]/College of American Pathologists [CAP] guidelines as the presence of 5% to 50% *ERBB2*-amplified cells by FISH or an area of tumor that is HER2 negative) (7) was the major determinant of pathologic complete response (pCR) to neoadjuvant T-DM1 and pertuzumab (T-DM1+P) treatment, with none of the HER2-heterogeneous cases having pCR (5). These results highlighted the importance of intratumor heterogeneity in treatment responses and the need to design improved treatment strategies for patients with heterogeneous tumors (8). Despite the importance of this issue, molecular and biological differences between HER2-heterogeneous and non-heterogeneous tumors have not been investigated.

Previous studies investigating T-DM1 treatment resistance mechanisms described multiple different preexisting and acquired alterations within tumor cells, including reduction of HER2 protein levels, as the primary reason for lack of response (9). Loss of HER2 protein can occur through downregulation of HER2 expression or its endocytosis, shifting the dependency toward other ERBB family members (10, 11). Mutations in *ERBB2* and components of its downstream signaling pathways, including loss of *PTEN*, *PIK3CA* activating mutations, and *YES1* amplification, have all been associated with resistance to T-DM1 (11–13). An immunosuppressive tumor microenvironment like high levels of regulatory T cells and PD-1 and PD-L1 immune checkpoint inhibitors also diminishes the efficacy of trastuzumab (14, 15). Lastly, upregulation of multi-drug-resistance transporters such as *ABCC1* could promote the efflux of DM1 from cells and confer resistance to T-DM1 (16).

In this study, we conducted transcriptomic profiling on 285 tissue samples, pretreatment biopsies (2 per tumor) and residual tumors, from 129 patients, along with *ERBB2* FISH and profiling of the immune microenvironment using NanoString digital spatial profiling. We investigated 3 main questions by integrated analyses of the data: (a) preexisting features associated with pCR versus no pCR, (b) treatment-induced changes in HER2 heterogeneity and transcription profiles, and (c) immune microenvironmental features associated with pCR versus no pCR and HER2 heterogeneity. To our knowledge, our study is the first to describe molecular features associated with intratumor HER2 and spatial heterogeneity and the relevance of these to response to neoadjuvant T-DM1+P treatment.

## Results

### Preexisting transcriptomic differences associated with HER2 heterogeneity and treatment response.

**Figure 1:**
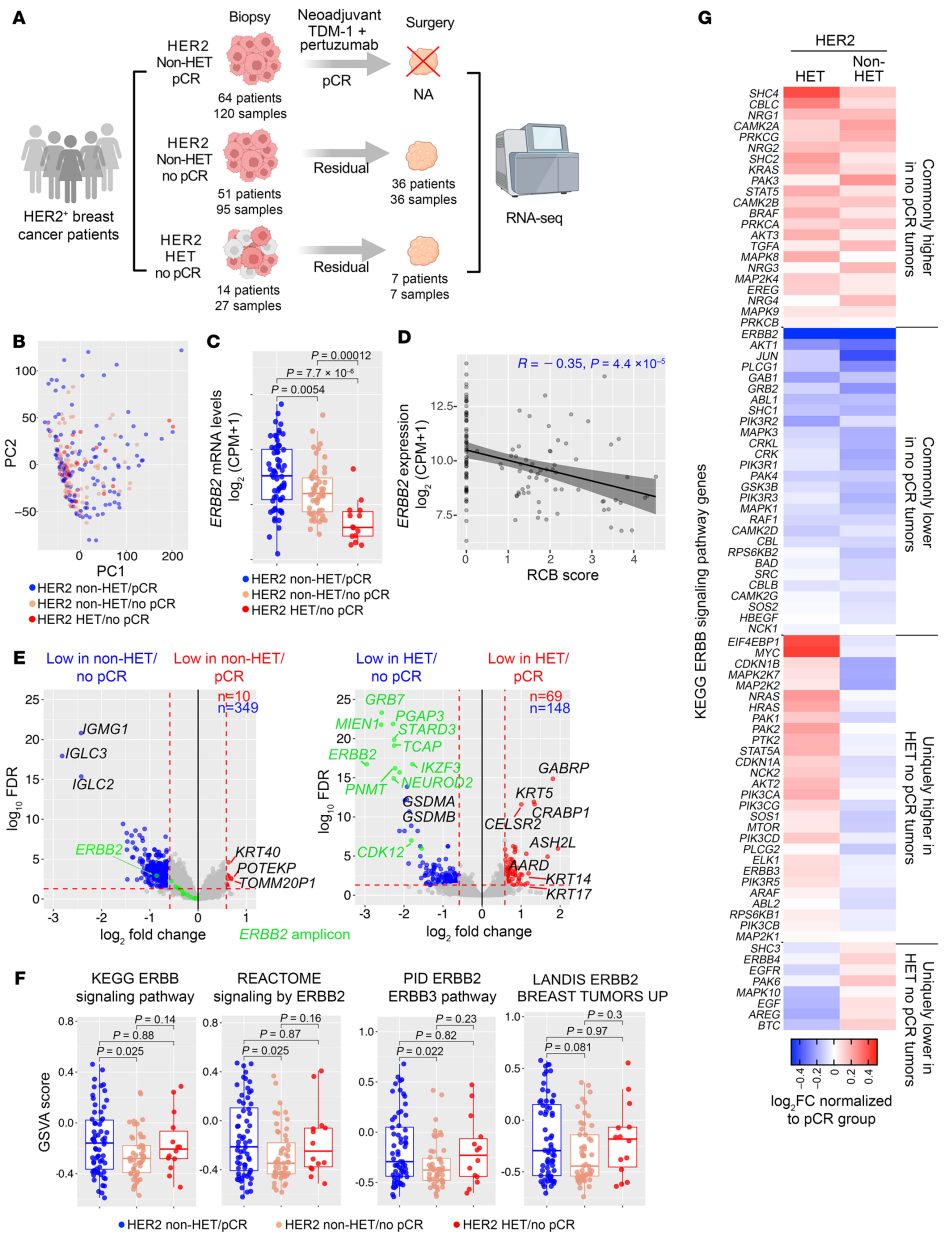
Transcriptional differences based on HER2 heterogeneity and response to treatment. (**A**) Schematic outline of the clinical trial and sample collection. (**B**) PCA plot depicting transcriptome variation of 242 pretreatment samples colored on the basis of HER2 heterogeneity and pCR status. (**C**) Box plots depicting *ERBB2* mRNA expression in the indicated groups at patient level using the mean expression of 2 biopsies from the same patient. (**D**) Scatterplot showing the correlation of RCB score and *ERBB2* expression in tumors assessed at patient level. (**E**) Volcano plots illustrating differentially expressed genes (DEGs) between pretreatment samples from the indicated comparisons. Top DEGs are indicated. (**F**) Box plots showing enrichment scores of 4 different ERBB/PI3K pathway signatures in HER2 non-HET/pCR, non-HET/no pCR, and HET/no pCR patients. Mean scores of 2 pretreatment biopsies from the same patient were used. (**G**) Heatmap depicting relative expression of KEGG ERBB signaling signature genes in HER2-HET and non-HET samples without pCR normalized to pCR samples. Genes are ordered from commonly different in the 2 groups compared with pCR cases to uniquely different in the HET and non-HET groups. *P* values were calculated based on 2-tailed Mann-Whitney *U* test (**C** and **F**) and Pearson’s correlation (**D**).

We performed RNA sequencing (RNA-Seq) on 292 pretreatment and posttreatment tissue samples composed of 2 spatially distinct research biopsies performed at baseline and residual tumors collected at the time of surgery from 129 patients enrolled in our previously reported phase II neoadjuvant T-DM1 plus pertuzumab clinical trial (5) (Figure 1A, Supplemental Table 1, and Supplemental Figure 1; supplemental material available online with this article; https://doi.org/10.1172/JCI176454DS1). Principal component analysis (PCA) identified treatment perturbation as a primary source of transcriptomic variation among all samples, and 7 biopsies were excluded from downstream analysis because of abnormal transcriptomic segregation (sample intrinsic variation outside the range of 5 × SD ± mean) (Supplemental Figure 2A). The mRNA levels of *ERBB2*, *ESR1*, and *PGR* showed significant positive correlation with corresponding protein levels scored by immunohistochemistry (IHC) performed on research biopsies, confirming the quality of the RNA-Seq data (Supplemental Figure 2, B and C). We classified patients into 3 subgroups based on pCR and HER2 heterogeneity (HET): HER2 non-HET/pCR (*n* = 64), HER2 non-HET/no pCR (*n* = 51), and HER2 HET/no pCR (*n* = 14). Of the 129 patients with tissue available for molecular profiling, 1 pretreatment biopsy was profiled for 16 cases (12.3%), and 2 pretreatment biopsies were profiled for 113 (87.7%). For the 43 patients profiled after treatment, only 1 sample was available (Figure 1A).

We first tested whether transcriptomic distances between 2 pretreatment biopsies from the same tumor were associated with any clinicopathologic features. PCA of 113 paired pretreatment biopsies showed a large degree of variation of transcriptomic distance within pairs (Supplemental Figure 2, D and E), but this was not associated with pCR and HER2 heterogeneity (Supplemental Figure 2F). The same observation was made when transcriptomic distances were correlated with quantitative parameters of treatment response (residual cancer burden [RCB] score) and the relative fraction of *ERBB2*-non-amplified cells (Supplemental Figure 2, G and H). Transcriptomic distance between 2 pretreatment biopsies from the same tumor was not associated with differences in tumor area, cellularity, patient age, and lymph node status but was significantly higher in ER-negative tumors (Supplemental Figure 2, I and J).

Next, we sought to identify potential mechanisms underlying preexisting resistance to T-DM1+P in HER2-HET and non-HET tumors. PCA of 242 pretreatment biopsies revealed a major homogenous cluster with a few deviations and no obvious associations with pCR or HER2 heterogeneity status, nor with other clinicopathologic features including tumor cellularity and lymph node and hormone receptor status (Figure 1B and Supplemental Figure 3A), suggesting that T-DM1+P resistance might be driven by specific pathways rather than global transcriptomic differences. Tumors derived from patients with pCR had the highest level of *ERBB2* expression, while HER2-HET tumors had the lowest (Figure 1C). *ERBB2* expression also inversely correlated with RCB score (Figure 1D), emphasizing the importance of HER2 abundance in determining response to T-DM1+P.

Differentially expressed genes (DEGs) between tumors from patients with pCR and no pCR varied depending on HER2 heterogeneity status (Figure 1E and Supplemental Table 2). Comparison of non-HET/pCR tumors with non-HET/no pCR revealed very few DEGs, except that non-HET/no pCR tumors had significantly lower expression of multiple immunoglobulin genes (e.g., *IGLC3* and *IGMG1*) (Figure 1E), implying potential differences in B cells. The topmost DEGs in the HET/no pCR versus non-HET/pCR comparison included genes located in the *ERBB2* amplicon (e.g., *ERBB2*, *GRB7*), along with pyroptosis mediators (*GSDMA* and *GSDMB*); both of these gene sets had higher expression in non-HET/pCR tumors. In contrast, genes with higher expression in HET/no pCR samples included basal cytokeratins (*KRT5*, *KRT14*, and *KRT17*), suggesting possible tumor subtype–related differences (Figure 1E). There was a limited overlap in DEGs between the 2 comparisons, mostly including genes with lower expression in no pCR relative to pCR tumors (regardless of HER2 heterogeneity) and again including genes in the *ERBB2* amplicon (e.g., *ERBB2*, *HOXB3*) and mesenchymal subtype–specific genes (e.g., *FN1*, *TNC*) (Supplemental Figure 3B), again highlighting the importance of *ERBB2* amplification and tumor subtype in response to T-DM1+P treatment.

Pathway enrichment analysis of DEGs identified FGFR signaling and bile acid metabolism as commonly high in no pCR tumors, while NOTCH signaling and ATF6 signaling pathways were commonly low (Supplemental Figure 3C). HET/no pCR tumors were enriched in mTOR signaling, DNA repair, cell cycle, and immune-related pathways, while very few pathways were uniquely enriched in non-HET/no pCR tumors (Supplemental Figure 3C). In line with differences in *ERBB2* expression, PI3K/ERBB signaling pathway signatures exhibited a lower level of enrichment in non-HET/no pCR tumors compared with that of non-HET/pCR tumors (Figure 1F), suggesting that reduced baseline activation might attenuate the dependency and thus alleviate the sensitivity toward HER2-targeted therapy in this subset. Intriguingly, however, HET/no pCR tumors displayed the same high level of PI3K/ERBB signature enrichment compared with the non-HET/pCR group despite a lower fraction of *ERBB2*-amplified cancer cells (Figure 1F). We hence hypothesized that other PI3K/ERBB pathway components besides HER2 might alternatively be overexpressed to sustain PI3K/ERBB pathway signaling in these tumors and explored this further. Comparing across 86 Kyoto Encyclopedia of Genes and Genomes (KEGG) ERBB signature genes, we found a subset of targets that were exclusively showing a higher trend in HET/no pCR tumors, including *MYC*, *NRAS*, and *EIF4EBP1* (Figure 1G and Supplemental Figure 3D), suggesting that these genes may alternatively sustain PI3K/ERBB signaling in HET/no pCR tumors. We also found that *ERBB4* had trends of higher expression in both HET/no pCR and non-HET/no pCR tumors compared with non-HET/pCR cases (Supplemental Figure 3D).

**Figure 2:**
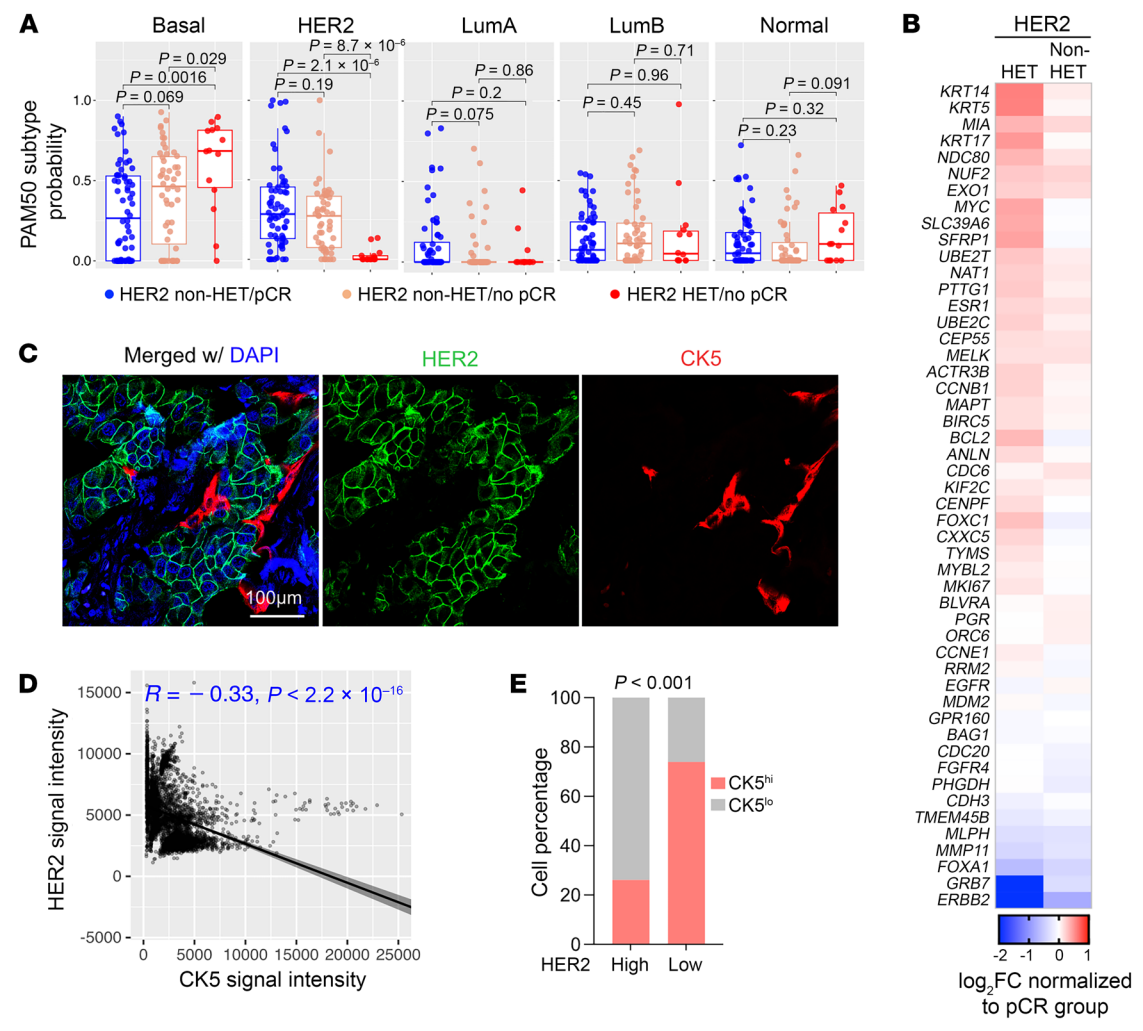
Intrinsic subtype correlation to HER2 heterogeneity and response. (**A**) Box plots depicting PAM50 subtype probability scores in the indicated groups using the mean scores of 2 biopsies from the same patient. (**B**) Heatmap showing relative expression of PAM50 genes in HET and non-HET samples without pCR normalized to pCR samples. (**C**) Representative immunofluorescence image of HER2 and cytokeratin 5 (CK5) in a HER2-HET tumor. Immunostaining was performed once. Scale bar: 100 μm. (**D**) Scatterplot representing correlation of HER2 and CK5 signal intensity in 4,367 cancer cells quantified from 8 tumors across all 3 subgroups. (**E**) Stacked bar plot depicting the percentage of CK5^hi^ and CK5^lo^ cancer cells within HER2^hi^ and HER2^lo^ cancer cell subpopulations. High and low were defined by median of all cells quantified. *P* values were calculated based on 2-tailed Mann-Whitney *U* test (**A**), Pearson’s correlation (**D**), and Fisher’s exact test (**E**).

Given the higher baseline expression of basal-like cytokeratins observed in HER2-HET tumors, we assessed the probability of each PAM50 subtype to explore potential subtype-specific differences. Remarkably, 93% (25/27) of HET biopsies were not classified as HER2-enriched subtype but rather as basal-like (22/27, 81.5%) or luminal A and B (LumA and LumB) subtype (5/27, 18.5%), regardless of hormone receptor status (Figure 2A and Supplemental Figure 3, E and F). In contrast, most non-HET/no pCR biopsies were still classified as HER2-enriched subtype (Figure 2A). The probabilities of basal or HER2 subtypes were either positively or negatively associated with RCB scores among all patients, indicating that a HER2-to-basal shift impacts treatment response (Supplemental Figure 3G). Further elaboration on the PAM50 gene panel revealed higher expression of several other basal subtype markers including *NDC80* and *SLC39A6* in addition to basal cytokeratins (e.g., *KRT5*) in HET tumors (Figure 2B). Immunofluorescence staining for HER2 and cytokeratin 5 (CK5) showed a mutual exclusivity pattern of HER2-high and CK5-high cells in these tumors (Figure 2, C–E). Overall, these findings suggest that HER2-HET tumors are more likely to have a basal-like intrinsic subtype while sustaining high levels of PI3K/ERBB signaling activation through upregulation of downstream pathway components.

### Treatment-induced changes in HER2 heterogeneity.

**Figure 3:**
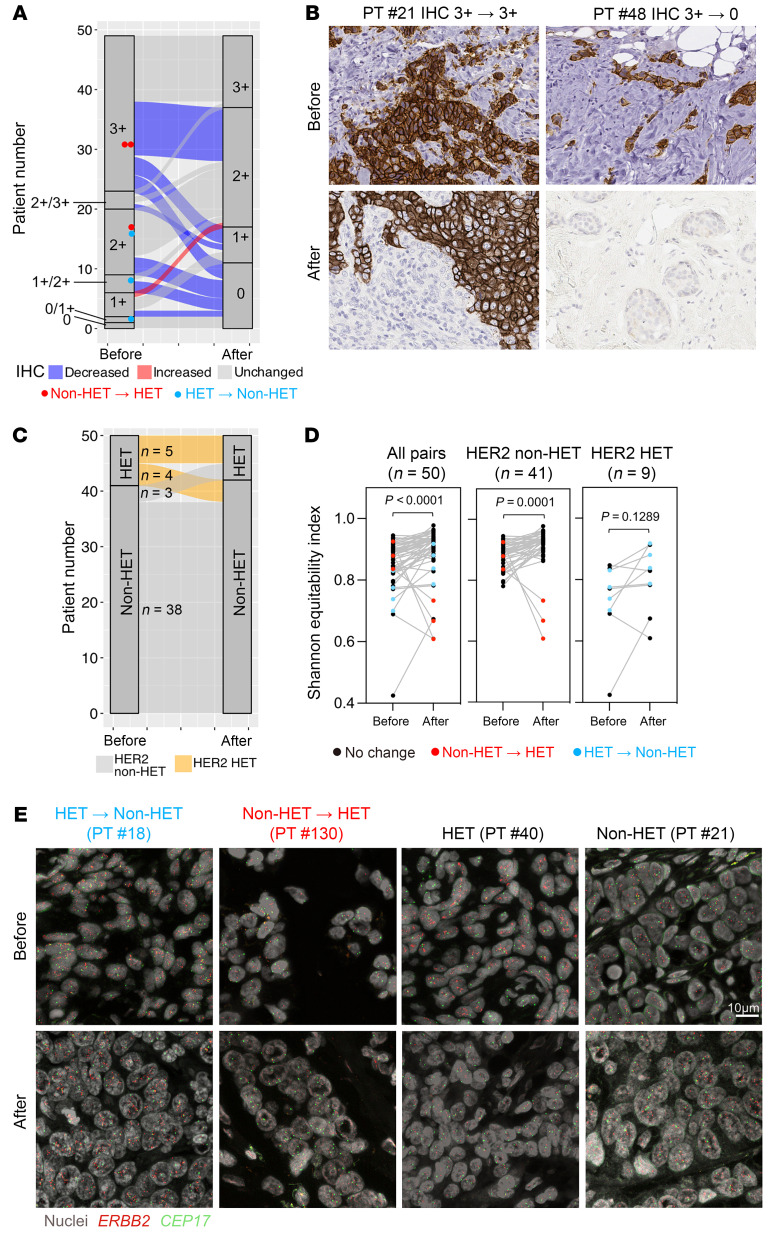
Treatment-induced changes in HER2 heterogeneity. (**A**) Sankey plot illustrating HER2 IHC score changes in pretreatment and posttreatment biopsies from 49 patients. Samples with HER2 heterogeneity shift are highlighted. (**B**) Representative images of intrapatient paired pretreatment and posttreatment HER2 IHC. Images were taken under ×40 magnification. (**C**) Sankey plot illustrating HER2 heterogeneity status shift in pretreatment and posttreatment biopsies from 50 patients. (**D**) Line plots depicting Shannon’s equitability index changes of *ERBB2* copy number in paired pretreatment and posttreatment biopsies among all or non-HET/HET patients. Samples with HER2 heterogeneity shift are highlighted. (**E**) Representative images of intrapatient paired pretreatment and posttreatment *ERBB2* FISH. Scale bar: 10 μm. Two-tailed Wilcoxon’s matched-pairs signed rank test (**D**).

To assess whether HER2 heterogeneity changes during treatment, we performed *ERBB2* FISH and HER2 IHC in 53 residual tumors from 53 patients (Supplemental Table 3). Two residual tumors failed both IHC and *ERBB2* FISH, 1 failed IHC, and 1 residual tumor’s paired pretreatment biopsy failed IHC and FISH, which resulted in 49 and 50 pairs of pretreatment and posttreatment samples for IHC and FISH comparative analyses, respectively. HER2 IHC scores among these 49 paired samples were lower in posttreatment compared with pretreatment tissues in nearly half of patients (26/49, 53.1%), while all but 1 of the rest (22/49, 44.9%) exhibited no change (Figure 3, A and B). HET tumors more commonly showed a decreasing trend in HER2 expression (5/9, 55.6%) compared with non-HET cases (17/40, 42.5%), and only 1 patient had an increase in HER2 IHC score in posttreatment tissue (Figure 3, A and B). HER2 heterogeneity in residual tumors was determined by FISH following ASCO/CAP guidelines, analyzing a median of 6 tumor areas per patient, with each area containing a median of 75 cells. We also quantified cellular diversity for *ERBB2* using single-cell copy number counts and Shannon’s equitability index as in our prior study (5). Residual tumors of patients that were HET had a distinctly lower fraction of *ERBB2*-amplified cells and lower *ERBB2*/*CEP17* copy number ratios compared with non-HET cases, which correlated with HER2 protein levels (Supplemental Figure 4, A and B). Tumors with high *ERBB2*/*CEP17* copy number ratio (≥6) had significantly greater cellular heterogeneity than ones with low (<6) in the pretreatment but not the posttreatment setting, suggesting that T-DM1+P treatment may preferentially target tumor cells with higher *ERBB2* copy number gain (Supplemental Figure 4C). Comparing pretreatment and posttreatment samples (*n* = 50 pairs), most non-HET tumors remained non-HET, while only 3 of 41 cases (7.3%) shifted to HET (Figure 3C). Conversely, 4 of 9 (44.4%) HET tumors were reclassified as non-HET following T-DM1+P treatment. Residual tumors had higher cellular *ERBB2* copy number heterogeneity regardless of pretreatment HER2 heterogeneity (Figure 3, D and E), *ERBB2* copy number gain, or hormone receptor status (Supplemental Figure 4, D and E). The 3 tumors that shifted from non-HET to HET showed a major decrease in Shannon’s equitability index (Figure 3D), the fraction of *ERBB2*-amplified cells, *ERBB2* copy number gain (Supplemental Figure 4F), and HER2 protein expression (Figure 3A), implying a selection for HER2-negative *ERBB2*-non-amplified cells during treatment. Furthermore, 3 of 4 samples that were HET and became non-HET exhibited an increase in the fraction of *ERBB2*-amplified cells but not *ERBB2* copy number gain (Supplemental Figure 4F) and became HER2-negative by IHC (Figure 3A). This result suggests either a possible disconnect between *ERBB2* copy number and HER2 protein levels due to the antibody used for IHC failing to detect certain truncated HER2 variants, or that there is a preexisting HER2-independent resistant mechanism, such as the coamplification of the 17q12 chemokine cluster with *ERBB2* that leads to an immunosuppressive microenvironment (17).

### Treatment-induced changes in transcriptomic profiles.

**Figure 4:**
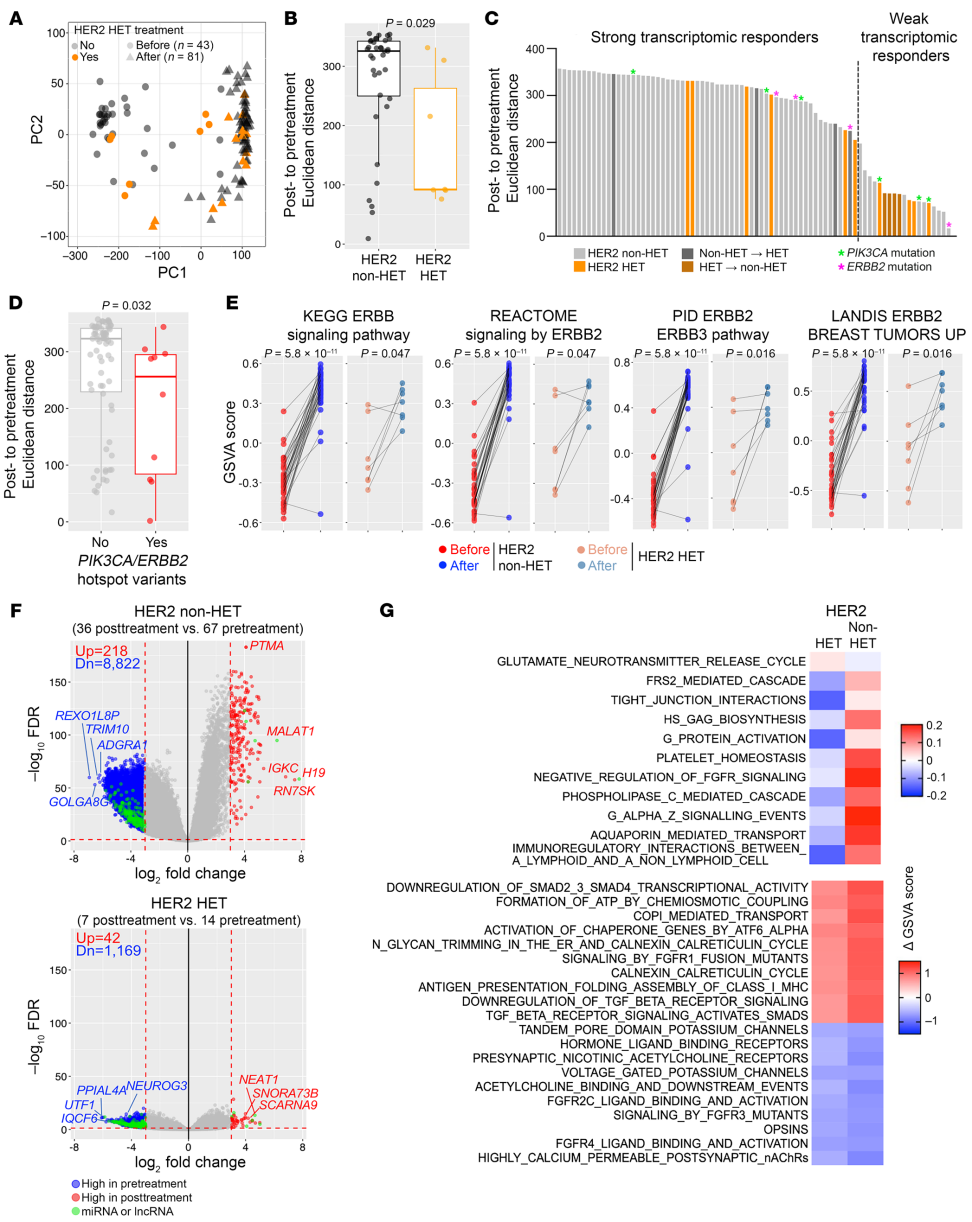
Treatment-induced changes in transcriptomic profiles. (**A**) PCA plot depicting transcriptomic variation among pretreatment (*n* = 81) and posttreatment (*n* = 43) biopsies. (**B**) Box plot showing Euclidean distances between pretreatment and posttreatment RNA-Seq samples in non-HET and HET patients. (**C**) Bar plot showing ranking of samples based on treatment-induced Euclidean distances. Pretreatment HET, HET shift, and *PIK3CA*/*ERBB2* mutation are indicated. (**D**) Box plot showing transcriptomic Euclidean distances between pretreatment and posttreatment samples in tumors with or without *PIK3CA* or *ERBB2* mutations. (**E**) Line plots showing enrichment scores of ERBB/PI3K pathway signatures in pretreatment and posttreatment samples of each patient. Mean scores of 2 biopsies were used to represent the pretreatment group of each patient. (**F**) Volcano plots illustrating DEGs between pretreatment and posttreatment biopsies from non-HET and HET samples. (**G**) Heatmap of top REACTOME pathways differentially enriched between pretreatment and posttreatment samples from non-HET and HET cases. Red and blue represent increased and decreased pathway enrichment, respectively, in posttreatment samples compared with pretreatment counterparts. Color scale represents magnitude of change. *P* values were calculated based on 2-tailed Mann-Whitney *U* test (**B** and **D**) and Wilcoxon’s matched-pairs signed-rank test (**E**).

We next investigated transcriptomic changes between pretreatment and posttreatment tumors in patients without pCR using RNA-Seq from 43 patients (81 pretreatment biopsies and 43 residual tumors). We observed a clear separation and greater transcriptomic distances between pretreatment and posttreatment samples among non-HET tumors, whereas minimal therapy-induced differences were detected in HET tumors (Figure 4, A and B). These treatment-associated transcriptomic changes were the most pronounced in tumors with a 3+ HER2 IHC score (Supplemental Figure 5A) and showed a positively correlated trend with the fraction of *ERBB2*-amplified cells in non-HET tumors (Supplemental Figure 5B), again emphasizing that the most important determinant of response to HER2-targeted therapy is the expression level of HER2. Intriguingly, HET tumors showed the opposite trend: the fraction of *ERBB2*-amplified cells negatively correlated with transcriptomic changes (Supplemental Figure 5B), suggesting that factors other than HER2 drive these treatment-induced changes. No significant associations were found between treatment-induced transcriptomic differences and patient age, hormone receptor status, and RCB scores (Supplemental Figure 5A), although the small sample size in some comparisons might limit the ability to see significant differences. It was also evident that treatment-induced changes were greater than differences between 2 biopsies from the same pretreatment tumor, and these 2 distances were not related (Supplemental Figure 5C). However, comparison of treatment-induced transcriptomic distances between paired intrapatient biopsies showed that tumors in older patients tended to display higher spatial heterogeneity in response (Supplemental Figure 5D).

Ranking of samples on the basis of Euclidean distances of pre-to-posttreatment transcriptomic changes illustrated a skewed distribution, implying divergent resistance mechanisms (Figure 4C). Therefore, we classified tumors into strong (top 75%) and weak (lowest 25%) transcriptomic responders and characterized their differences in more detail (Figure 4C). Changes in HER2 heterogeneity between pretreatment and posttreatment samples were not significantly associated with the extent of transcriptomic response, although all tumors that switched from HET to non-HET had a weak response (Figure 4C). Among HET tumors, patients with older age and higher RCB score tended to have stronger transcriptomic response (Supplemental Figure 5E). These tumors also displayed higher cellular heterogeneity for *ERBB2*, lower fraction of *ERBB2*-amplified cells, and lower *ERBB2* copy number gain, although the sample size was limited (Supplemental Figure 5E). In non-HET tumors, the degree of transcriptomic response did not correlate with RCB score, patient age, and *ERBB2* copy number changes (Supplemental Figure 5F).

### Potential mechanisms of treatment resistance.

Mutations in *PIK3CA* and *ERBB2* have been identified as prominent drivers of resistance to HER2-targeting therapies (18). Thus, we analyzed our RNA-Seq data for known mutational hotspots in *PIK3CA* (exons 5, 8, 10, and 21) and *ERBB2* (exons 11, 22, and 23) (Supplemental Table 4). We identified 17 (13.5%) *PIK3CA*-mutant and 4 (3.2%) *ERBB2*-mutant tumors; the majority (70.6%) of *PIK3CA* mutations occurred at residue H1047, and all 4 *ERBB2* variants were at V777 residue (Supplemental Figure 5, G and H). Analysis of the frequency of these mutations in multiple biopsies from the same patient, pre-to-posttreatment samples, or associations with pCR, hormone receptor, and HER2 heterogeneity status did not reveal any significant differences, although most mutations were already present before treatment (Supplemental Figure 5, I–K). Tumors with *PIK3CA* or *ERBB2* mutations exhibited smaller pre-to-posttranscriptomic distances, suggesting that these alterations are mechanisms of de novo resistance (Figure 4D).

To further explore associations between PI3K/ERBB pathway and transcriptomic changes, we analyzed the expression of pathway components in pretreatment and posttreatment tumors at patient level. In non-HET cases, we observed increased expression of *ERBB2* and *ERBB3* but decreased expression of *ERBB4* in residual tumors, whereas no changes were observed in HET tumors (Supplemental Figure 6, A and B). Enrichment scores of PI3K/ERBB pathway signatures were augmented in posttreatment samples regardless of preexisting HER2 heterogeneity and the degree of transcriptomic response strength, implying upregulation of compensatory mechanisms downstream of HER2 (Figure 4E and Supplemental Figure 6, A and B).

Next, we performed DEG analyses between pretreatment and posttreatment samples to characterize global changes in transcriptomes. Non-HET tumors exhibited significantly more DEGs (adjusted *P* value < 0.05 and |fold change| > 8) compared with HET tumors even after controlling for differences in sample size (Figure 4F, Supplemental Figure 6C, and Supplemental Table 2). A subset of DEGs, mostly encoding microRNAs and long noncoding RNAs, in HET tumors were also detected in non-HET cases (Figure 4F and Supplemental Figure 6D). Pathway enrichment analysis uncovered TGF-β, FGFR, mTOR, PTEN, and neurotransmitter signaling as commonly altered in posttreatment tumors regardless of HER2 heterogeneity. More unique treatment-induced pathway changes were detected in non-HET tumors compared with HET tumors, including pathways related to protein glycosylation, G protein activation, and steroid biosynthesis (Figure 4G and Supplemental Figure 6E). Lastly, we observed a switch in tumor subtype from basal-like to a more LumA-like profile regardless of HER2 heterogeneity (Supplemental Figure 6F).

In conclusion, our RNA-Seq analyses revealed significant global transcriptomic differences between pretreatment and posttreatment tumors related to HER2 heterogeneity in patients without pCR. While PI3K/ERBB signaling activation emerged as a prominent mechanism of resistance regardless of HER2 heterogeneity, distinct transcriptomic responses indicate the presence of multiple different mechanisms of resistance.

### Preexisting differences in the tumor immune microenvironment.

**Figure 5:**
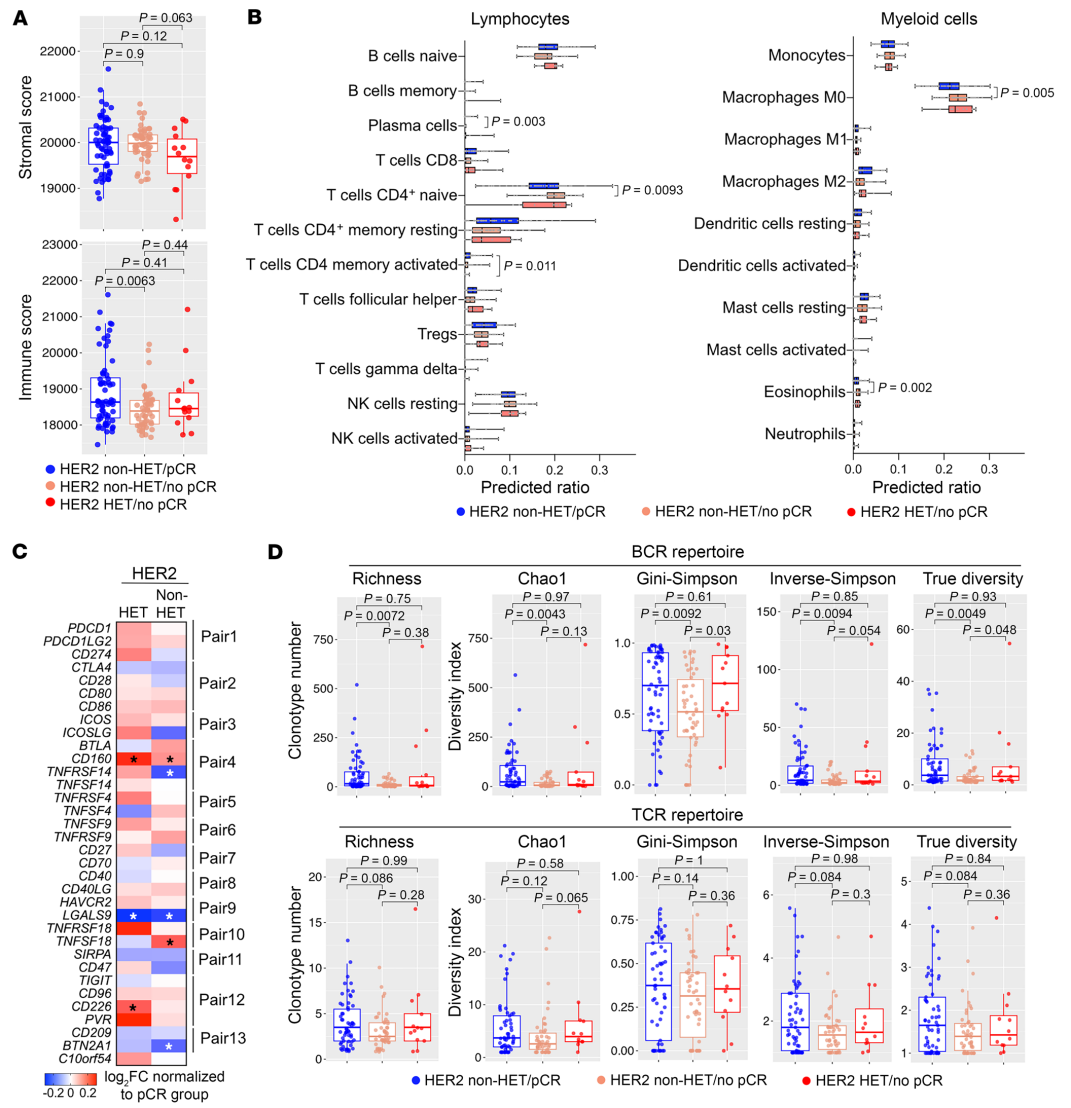
Preexisting immune microenvironmental differences and treatment response. (**A**) Box plots depicting predicted overall stromal and immune scores in the indicated patient groups. Mean scores of the 2 pretreatment biopsies from the same patient were used. (**B**) Box plots showing predicted abundances of lymphocyte and myeloid cell subsets from the 2 pretreatment biopsies from the same patient in the indicated groups. (**C**) Heatmap showing relative expression of immune checkpoint genes in samples without pCR normalized to pCR cases. Asterisks indicate statistically significant (*P* < 0.05) differences. (**D**) Box plots depicting BCR and TCR richness and diversity index scores in the indicated groups. Average measurements of the 2 pretreatment biopsies from the same patient were used for pretreatment values. *P* values were calculated based on 2-tailed Mann-Whitney *U* test in all panels.

Immune cells play key roles in response to HER2-targeted therapies (19). Thus, we investigated potential differences in the tumor immune environment according to treatment response and HER2 heterogeneity. Using the ESTIMATE algorithm (20), we assessed the proportions of immune and stromal components in treatment-naive tumors. Compared with tumors from pCR patients, non-HET/no pCR cases had significantly lower immune scores, while stromal scores showed a lower trend in HET/no pCR tumors compared with non-HET cases (Figure 5A). Hormone receptor–positive (HR-positive) and HR-negative tumors had opposing immune profiles, which may have dampened the observed differences between pCR and no pCR cases (Supplemental Figure 7, A and B). Immune and RCB scores showed significant inverse correlation, highlighting the importance of the preexisting tumor immune microenvironment in determining treatment response (Supplemental Figure 7B). Prediction of immune cell subtypes using CIBERSORT (21) showed that HET/no pCR tumors had a significantly lower proportion of activated CD4^+^ memory T cells than the pCR group, whereas significantly lower plasma cells but higher CD4^+^ naive T cells and M0 macrophages were predicted in non-HET/no pCR tumors (Figure 5B). HET/no pCR tumors also more commonly exhibited higher expression of immune checkpoint receptor-ligand pairs including *CD274* (PD-L1), *CD160*, and *CD226*-*PVR* compared with non-HET/no pCR tumors (Figure 5C and Supplemental Figure 7C).

Next, we interrogated preexisting T and B cell immune repertoire differences among the 3 groups of pretreatment tumors by inferring T cell receptor (TCR) and B cell receptor (BCR) clonotypes using the TRUST4 algorithm (22) applied to our RNA-Seq data. BCR clonal abundance was overall much higher than TCR (Supplemental Figure 7D). Non-HET/no pCR tumors showed the lowest BCR richness and diversity compared with both non-HET/pCR and HET/no pCR tumors (Figure 5D and Supplemental Figure 7D), consistent with our RNA-Seq data showing significantly lower expression of multiple immunoglobulin genes in non-HET/no pCR compared with non-HET/pCR tumors (Figure 1E). Differences in TCR richness and diversity were much less pronounced among tumors, although non-HET/no pCR tumors again had slightly lower values (Figure 5D and Supplemental Figure 7D).

**Figure 6:**
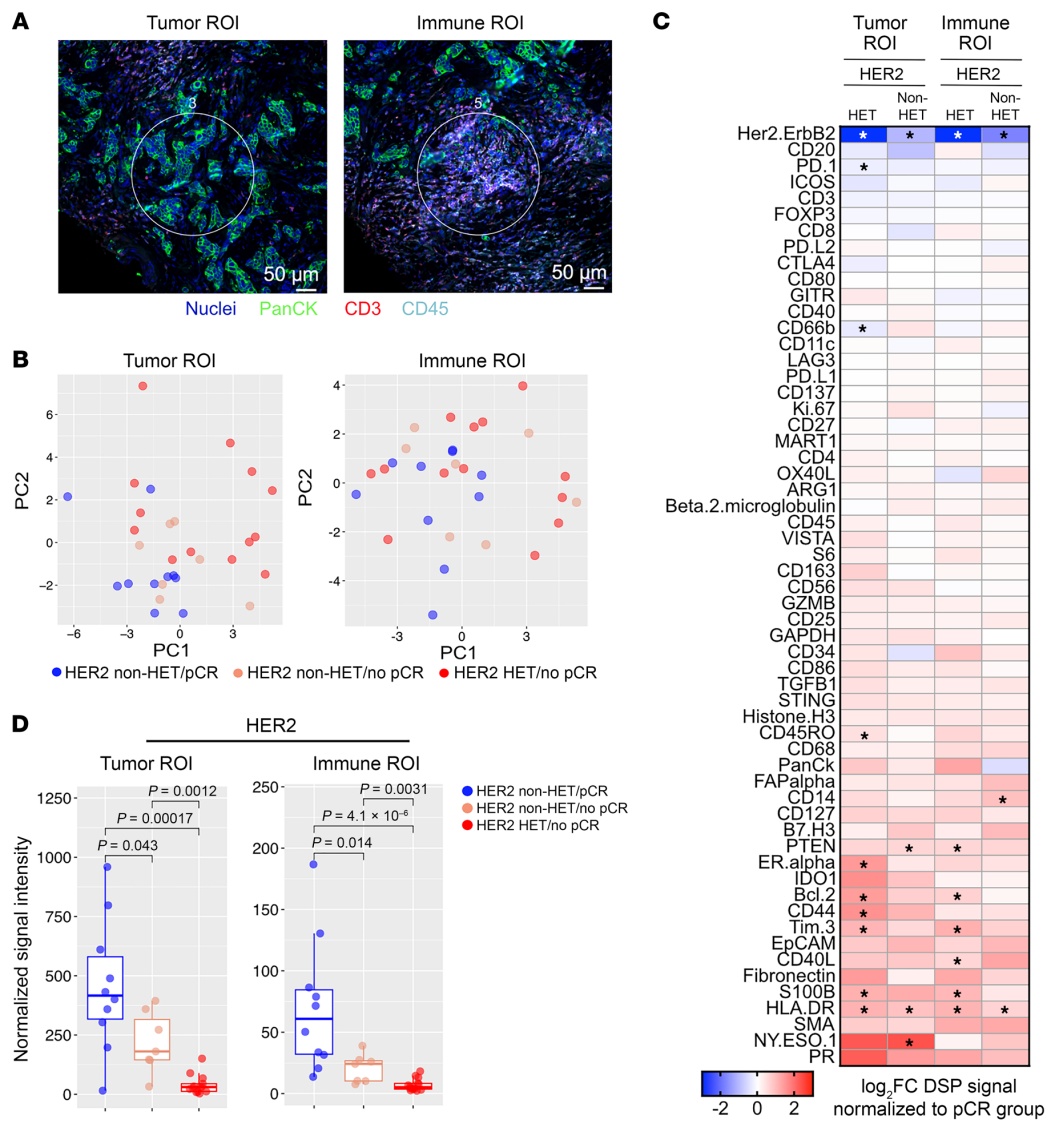
Digital spatial profiling of the immune environment. (**A**) Representative images of digital spatial profiling (DSP) of tumor and immune regions of interest (ROIs). Scale bars: 50 μm. (**B**) PCA plot depicting the DSP signal variation from tumor and immune ROIs of the 58 target protein expression included in the DSP panel among 30 pretreatment samples of the indicated patient groups. (**C**) Heatmaps showing relative expression of 58 proteins by DSP in samples without pCR normalized to pCR samples. Asterisks indicate statistically significant (*P* < 0.05) differences. Comparisons were separated into tumor and immune ROIs. (**D**) Box plots showing the normalized expression of HER2 among the 3 groups in pretreatment samples in both tumor and immune ROIs. *P* values were calculated based on 2-tailed Mann-Whitney *U* test (**C** and **D**).

To comprehensively explore the differences in the immune microenvironment at the protein level, we used NanoString digital spatial profiling (DSP) with 61 targets on a subset of 62 samples from 30 patients with matched RNA-Seq data. Regions of interest (ROIs) for tumor epithelial and immune areas were first defined based on pan-cytokeratin epithelial and CD3 and CD45 immune cell markers (Figure 6A). A median of 4 tumor epithelial and 2 immune ROIs were scored for each biopsy. The immune and tumor epithelial ROIs were clearly separated, with distinct enrichment of epithelial (e.g., EpCAM, PanCK) and immune (CD3, CD45) cell markers, whereas mouse IgG2a antibody (negative control) showed minimal signal (Supplemental Figure 7E). In addition, significant positive correlation between mRNA and protein levels was observed for cell type–specific markers in both tumor and immune ROIs (Supplemental Figure 7F), confirming the quality of the DSP data. PCA on pretreatment samples showed better separation of pCR and no pCR samples at tumor but not immune ROIs (Figure 6B). Comparison of baseline target expression in pCR and no pCR tumors separated by HER2 heterogeneity status again demonstrated distinct enrichment patterns in these 2 tumor subsets (Figure 6C). HER2 protein levels were significantly lower in no pCR compared with pCR cases in both tumor and immune ROIs with a more pronounced difference in HET tumors (Figure 6D). In contrast, HLA-DR, an MHC-II class protein, showed higher expression in no pCR compared with pCR tumors (Supplemental Figure 7G). Several targets showed specifically higher expression in HET cases, including TIM3, BCL2, S100B, and CD44 (tumor ROI only), implying a more immunosuppressive microenvironment within these tumors (Supplemental Figure 7G).

Overall, these data strongly support a role for a more immunosuppressive environment contributing to resistance to treatment in both non-HET/no pCR and HET tumors, but through different mechanisms. Non-HET/no pCR tumors tend to have fewer adaptive immune cells, while HET/no pCR tumors display an immune activation/exhaustion state marked by higher expression of several immune checkpoint proteins.

### Immune microenvironmental differences between pretreatment and posttreatment tumors.

**Figure 7:**
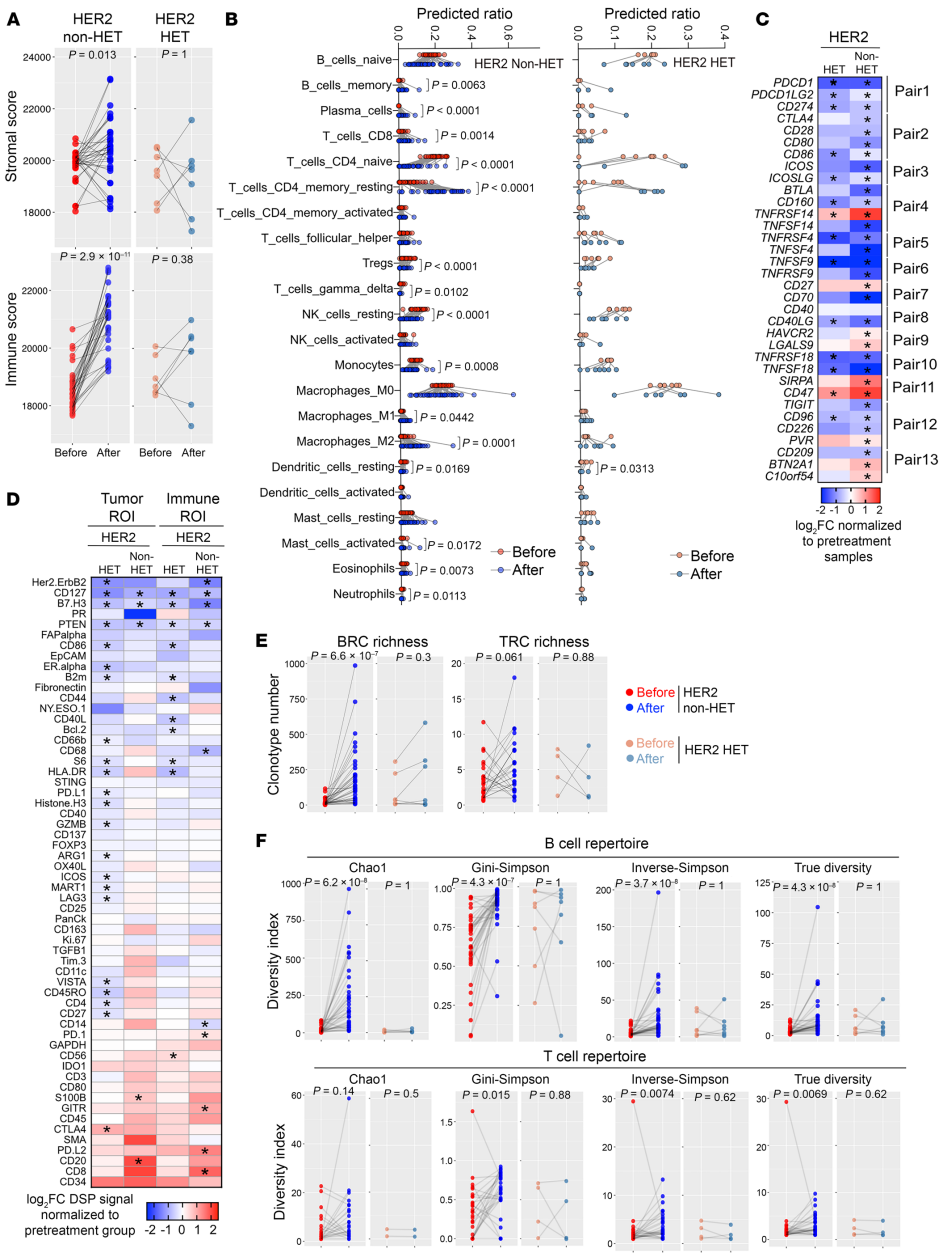
Treatment-induced changes in the immune environment. (**A**) Line plots depicting predicted overall stromal and immune scores among the 4 subgroups based on HER2 heterogeneity and pre-to-posttreatment samples from the same patient. Mean scores of 2 biopsies were used to represent the pretreatment group of each patient. (**B**) Line plots showing predicted lymphocyte and myeloid cell subset abundances in the indicated pre-to-posttreatment pairs. Average ratio of 2 biopsies was used to represent the pretreatment sample of each patient. (**C** and **D**) Heatmaps showing treatment-induced changes in the expression of immune checkpoint genes (**C**) and 58 proteins used in digital spatial profiling (**D**). Asterisks indicate statistically significant (*P* < 0.05) differences. In **D**, comparisons are separated into tumor and immune ROIs. (**E** and **F**) Line plots depicting BCR and TCR richness (**E**) and diversity index scores (**F**) in the indicated pre-to-posttreatment pairs. Mean measurements of 2 biopsies were used to represent the pretreatment sample of each patient. *P* values were calculated based on 2-tailed Wilcoxon’s matched-pairs signed-rank test in all panels.

Lastly, we explored differences in the immune microenvironment between pretreatment and posttreatment samples of patients without pCR. We detected a significant increase in the proportion of immune and stromal components after treatment only in tumors from non-HET cases (Figure 7A). In line with this, CIBERSORT predicted an increase in the relative numbers of activated immune cell subtypes in non-HET tumors including CD8^+^ T cells, CD4^+^ memory T cells, memory B cells, plasma cells, activated mast cells, and M1-like macrophages and a decrease in regulatory T cells and CD4^+^ naive T cells (Figure 7B). In contrast, HET tumor showed minimal changes in immune cell infiltration and composition after treatment despite a significant decrease in the levels of most immune checkpoint receptor-ligand pairs in both HET and non-HET tumors (Figure 7C). Only *TNFRSF14* and *CD47* had higher levels in posttreatment samples (Figure 7C).

We further elaborated these findings by analyzing NanoString DSP results of 19 pairs of pretreatment and posttreatment biopsies with 6 pairs of non-HET and 13 pairs of HET tumors (Figure 7D). HER2 protein expression was significantly lower in residual tumors compared with matched pretreatment samples (Supplemental Figure 8A), in line with the IHC results (Figure 3A). Several active immune markers were uniquely elevated in non-HET posttreatment tumors, including CD20 (tumor ROI) and CD8 (immune ROI), indicating increased CD8^+^ T cell and B cell infiltration after treatment (Supplemental Figure 8B). In contrast, other immune activation markers including CD86, CD44, CD40L, HLA-DR, and granzyme B were significantly lower in HET posttreatment relative to pretreatment tumors, while CD127 and PTEN were universally downregulated in all the groups (Supplemental Figure 8B). This decrease in PTEN might contribute to the enrichment of PIK3CA/ERBB2 signatures in residual tumors.

In an analysis of T and B cell repertoire of paired samples from RNA-Seq, we found a strong increase in richness and diversity of BCRs in non-HET residual tumors and no changes in HET tumors, although these had a higher level of BCR richness and diversity in pretreatment tumors (Figure 7, E and F).

## Discussion

HER2-targeting antibody-drug conjugates (ADCs), including T-DM1, have demonstrated remarkable efficacy in HER2-positive breast cancer, leading to significant improvements in clinical outcomes. Despite this success, resistance to HER2-targeting ADCs remains a major challenge, and overcoming this requires the molecular understanding of underlying mechanisms. In the present study, we performed multilevel profiling of pretreatment and posttreatment samples from a phase II neoadjuvant clinical trial using T-DM1 and pertuzumab and identified potential genetic, transcriptomic, and microenvironmental mechanisms of resistance. Importantly, our study is, to our knowledge, the first in-depth characterization of HER2-HET and non-HET tumors, and the differences we identified could serve as a basis for the design of more effective individualized treatment strategies. Several preexisting features were commonly associated with T-DM1+P resistance, including lower *ERBB2* expression and a HER2-to-basal intrinsic subtype shift, whereas lower HER2 signaling pathway activation, immune infiltration, and immune repertoire abundance were specific to the non-HET/no pCR group. Interrogation of treatment-induced differences revealed that HER2-HET tumors exhibited minimal transcriptomic response, while non-HET tumors showed increased ERBB signaling and immune activation. Key findings of our study are summarized in Supplemental Table 5.

We initially investigated preexisting mechanisms driving T-DM1+P resistance in HER2-HET and non-HET tumors by analyzing differences in pretreatment biopsies. We found that high *ERBB2* expression was the most prominent feature positively associated with pCR status, which is consistent with recent findings in the phase III KRISTINE neoadjuvant trial comparing T-DM1+P versus docetaxel + carboplatin + trastuzumab + pertuzumab in patients with HER2-positive stage II–III breast cancer (23). Similarly, in the TH3RESA trial comparing T-DM1 versus treatment of physician’s choice (TPC) in previously treated HER2-positive advanced-stage tumors, the progression-free survival benefit favoring T-DM1 over TPC was greater in cases with higher *ERBB2* mRNA levels (24). Higher HER2 protein expression was also a strong predictor of pCR and progression-free survival in the neoadjuvant phase III NEOALTTO study testing the efficacy of lapatinib and trastuzumab combination (25). In contrast, in the NSABP B-47/NRG Oncology clinical trial testing whether addition of trastuzumab to adjuvant chemotherapy would improve invasive disease-free survival (IDFS) in patients with *ERBB2*-non-amplified, HER2 IHC 1+ or 2+ high-risk invasive breast cancer, trastuzumab did not prolong IDFS regardless of HER2 expression level (26). These results suggest important biological differences between *ERBB2*-amplified and -non-amplified tumors and between HER2-HET and HER2-low cases. They also highlight the importance of molecular understanding of the distinct evolutionary paths of different HER2-positive tumor subtypes for optimal treatment design.

Despite lower overall *ERBB2* expression, HER2-HET tumors retained levels of PI3K/ERBB pathway activation equivalent to those in non-HET/pCR cancers, likely due to increased expression of downstream pathway components including *MYC* and *EIF4EBP1*. Comprehensive single-cell profiling of *ERBB2*-amplified and -non-amplified cells within the same HER2-HET tumor would be required to understand the source of this compensatory mechanism. We also observed uniquely lower expression of pyroptosis mediators (*GSDMA* and *GSDMB*) in HER2-HET tumors compared with pCR samples, suggesting perturbed apoptotic and innate immune signaling pathways as potential mechanisms of treatment resistance (27). Conversely, non-HET tumors resistant to T-DM1+P had lower *ERBB2* expression and weaker PI3K/ERBB pathway activation, suggesting decreased dependence on this pathway and insensitivity to anti-HER2 agents. Intriguingly, neurotransmitter pathways such as olfactory signaling and dopamine release cycle were exclusively enriched in this tumor subset, implying alternative molecular dependencies for cell survival. For instance, previous studies have uncovered a role of dopamine and serotonin in promoting cancer cell proliferation and modulating immune responses to allow cells to evade chemotherapy (28). Further investigation is warranted to explore these tantalizing findings.

By quantifying T-DM1+P–induced transcriptional changes, we observed minimal transcriptomic responses in HET tumors despite the presence of HER2-positive cancer cells, implying key biological differences between HER2-HET and non-HET tumors. For example, *ERBB2*-amplified and -non-amplified cells might functionally interact with each other directly or indirectly, diminishing response to T-DM1+P. Additionally, the decrease in HER2 protein expression during treatment will reduce T-DM1+P binding, diminishing its efficacy. While deciphering biological differences between HER2-HET and non-HET tumors requires further studies, our results clearly prove the limited utility of HER2-targeting antibodies in HET tumors and highlight the need to stratify patients based on HER2-HET status when deciding on treatment strategies. Specifically, patients with HET tumors likely benefit from combination therapies that include chemotherapeutic and immunomodulatory agents. In addition, small-molecule inhibitors of ERBB family members like lapatinib and neratinib and targeting of downstream components of the PI3K/AKT signaling pathway including mTOR and MYC may be more effective in this subset of patients than HER2 antibody–based approaches. Future randomized clinical trials would be required to define optimal treatment strategies for HER2-HET tumors.

Prior data showed that subclones with *PIK3CA* or *ERBB2* hotspot missense mutations are dominantly selected in treatment-resistant HER2-positive tumors as a result of PI3K pathway hyperactivation (29–31). Similarly, in the KRISTINE trial, *PIK3CA* mutations were associated with a lower rate of pCR in the T-DM1+P treatment group (23). Although we did not observe any association of *PIK3CA* exon 9 and exon 20 hotspot mutations (called from RNA-Seq) with pCR, we found weaker treatment-induced transcriptomic changes in *PIK3CA-*mutant tumors, likely due to the large fraction of these preexisting mutant clones. The greater transcriptomic response in tumors without *PIK3CA* and *ERBB2* hotspot mutations could be due to selection for a preexisting rare subpopulation or a significant shift in cell state. We also identified 3 residual tumors that shifted from non-HET to HET status based on *ERBB2* FISH, accompanied by a dramatic decrease of *ERBB2*-amplified cell fraction. This shift likely resulted from the selection for resistance-conferring dominant subclones without *ERBB2* amplification. For instance, one of these shifted tumors harbored an *ERBB2* V777L activating mutation, eliminating the selective pressure for *ERBB2* amplification. However, it is also plausible that the pretreatment biopsies of these cases missed the region of the tumor with *ERBB2*-non-amplified cells.

We also investigated pCR and HER2-HET status–related differences in the immune microenvironment. Compared with non-HET/pCR samples, non-HET/no pCR tumors exhibited a more immunosuppressive microenvironment before treatment, which might have contributed to their muted response to T-DM1+P. On the other hand, HER2-HET tumors, none of which achieved pCR, showed immune exhaustion at baseline, with higher expression of immune checkpoint proteins like TIM3. Elevated TIM3 levels may hinder cytotoxic T cell activity in these tumors without affecting overall immune infiltration, offering a potentially actionable therapeutic opportunity using TIM3-blocking antibodies like sabatolimab (32). T-DM1+P treatment induced a more immunosuppressive microenvironment in HET tumors, potentially contributing to lack of treatment response. These findings highlight the importance of evaluating the impact of systemic therapies on the tumor microenvironment at different time points during treatment. Combining T-DM1 with immunomodulatory agents might enhance antitumor immune responses while simultaneously targeting HER2-positive cancer cells.

Our study has provided valuable mechanistic insights into therapeutic resistance driven by HER2 heterogeneity, although it has certain limitations. First, the study employed numerous exploratory analyses, and caution is needed when interpreting some of the results, even when numeric and statistical differences are observed, owing to the risk of false discoveries. Validation in independent studies using the same treatment and methodologies would be required to strengthen the conclusions. Second, pCR reflects the response of the primary tumor to short-term treatment. Reassessment of the patients after long-term follow-up would be necessary to determine the impact of HER2 heterogeneity on disease-free and overall survival. However, the non-uniform treatment of this patient cohort during adjuvant therapy makes such analyses challenging. Third, our study design did not include single-agent treatment arms, making it impossible to define the impact of HER2 heterogeneity on response to T-DM1 and pertuzumab individually. Although both agents target HER2, resistance mechanisms may be distinct. For example, the preexisting higher cell cycle signature observed in HET tumors might play a more dominant role in resistance toward the cytotoxic payload of T-DM1. On the other hand, the preexisting immune exhaustion features such as higher TIM3 and higher MHC expression are likely to be associated with pertuzumab resistance as previously found in the NeoSphere trial (33). Lastly, we had a few cases with discordant levels of HER2 defined by RNA, FISH, and protein measurements. For example, HER2 IHC scores did not always align with *ERBB2* mRNA levels, potentially owing to the antibody that was generated against a synthetic C-terminal human ERBB2 fragment missing certain HER2 protein variants. Furthermore, intratumor heterogeneity could also contribute to these differences, as the specimens used for different assays were obtained from different sections of the same biopsy. In line with this, the diagnostic and research biopsies did not have the same IHC scores for ER and PR in a few cases.

Overall, investigating the molecular mechanisms of T-DM1+P resistance in HER2-positive breast cancer holds immense promise in augmenting our understanding of the disease and improving treatment outcomes. Our study provides fundamental clinical evidence supporting distinct resistance mechanisms in HET and non-HET tumors from both cell-autonomous and non-cell-autonomous sources, potentially paving the way to new, personalized therapeutic opportunities and ultimately bringing us closer to the effective treatment of all patients with HER2-positive breast cancer.

## Methods

### Sex as a biological variable.

Breast cancer predominantly affects women, with approximately 99% of cases occurring in females. Therefore, only female patients were included in this study, and the findings are not applicable to males.

### Tumor cellularity assessment.

Tumor cellularity was measured as previously described (34). Hematoxylin and eosin–stained (H&E-stained) histologic sections from both core needle biopsies and subsequent resection specimens were studied. Cellularity was calculated per square millimeter as the percentage of tissue area that was occupied by invasive tumor cells.

### ERBB2 FISH and heterogeneity assessment.

Central pathology evaluation of HER2 status was performed at the European Institute of Oncology in Milan. Formalin-fixed, paraffin-embedded (FFPE) tumor tissue sections of 53 patients were stained with H&E to confirm the presence of residual tumor, and immunostained for HER2 using the HercepTest kit (SK001, Dako/Agilent) according to the manufacturer’s instructions. The polyclonal HercepTest Rabbit Anti-Human HER2 Protein Antibody was generated using a synthetic C-terminal fragment of human HER2 protein (intracellular part) as immunogen. Immunostaining results were scored according to the current ASCO/CAP scoring system, and the percentage of tumor cells exhibiting each individual score (from 0 to 3+) was recorded, together with the spatial distribution (focal or diffuse) of cells with negative or equivocal (2+) scores. *ERBB2* copy number status was assessed by fluorescence in situ hybridization (FISH) using the HER2 IQFISH pharmDx assay (Dako/Agilent) according to the manufacturer’s instructions. FISH-stained slides were evaluated at a Zeiss Axio Imager Z2 microscope, equipped with Metafer 4 Metacyte (MetaSystems) software for automating microscopy and processing microscopy images. Each slide was evaluated visually and independently by 2 trained readers counting at least 60 tumor cells, and subsequently by the Metafer software counting an average number of approximately 300 tumor cells. HER2 heterogeneity was evaluated following the recommendations of the College of American Pathologists and the United Kingdom HER2 testing guidelines. HER2 heterogeneity was defined as the existence of a population of tumor cells with *ERBB2* amplification (i.e., *ERBB2*/CEP17 ratio ≥ 2.0 or a gene copy number ≥ 6) representing more than 5% but less than 50% of tumor cells. HER2 immunostaining and *ERBB2* FISH were compared for each case to ensure that any immunohistochemically heterogeneous area of the tumors had been evaluated in the FISH preparations.

Shannon’s equitability index was used to quantify *ERBB2* heterogeneity following our previous study (5). Briefly, for each patient *i*, where *i* = 1, 2, … *n*, we obtained the *ERBB2*, *x_ik_*, and CEP17, *y_ik_*, levels for each cell *k* = 1, … *K* in all the images from the same patient. Each cell was then denoted by the tuple (*x_ik_* = *h*, *y_ik_* = *c*), where *h* = 1, 2, … *H* is the *ERBB2* level and *c* = 1, 2, … *C* is the CEP17 copy number. We then calculated the frequency of each tuple in each patient, where *Z_ihc_* is the frequency of (*h*, *c*) cells in patient *i* and |*Z_i_*| is the total number of species from patient *i*. Each species then has frequency *p_ihc_* = *Z_ihc_*/|*Z_i_*|. Finally, we calculated the Shannon index for each patient: *H_i_* = –Σ(*h*,*c*)*p_ihc_*ln(*p_ihc_*); and the Shannon equitability index: *E_i_* = *H_i_*/ln(|*Z_i_*|). We used the fraction of *ERBB2-*amplified cells to assess the level of *ERBB2* amplification within each patient. A cell was considered amplified if the *ERBB2*/CEP17 ratio was ≥2 or the gene copy number was ≥6. The fraction of *ERBB2-*amplified cells was calculated to be the percentage of amplified cells out of the entire population.

### Immunofluorescence staining.

Tissue sections were baked overnight at 60°C, deparaffinized with xylene, and rehydrated using a gradient of ethanol. Antigen retrieval was performed using Target Retrieval Solution, pH 6 (Agilent), for 20 minutes in a steamer. Sections were blocked with TBST 5% goat serum and then incubated with primary antibodies overnight at 4°C (HER2; 1:200; ab16901, Abcam) and anti–cytokeratin 5 antibody (1:200; ab52635, Abcam). Antibodies were pre-conjugated with Alexa Fluor 488 (HER2) and Alexa Fluor 555 (KRT5) using the Abcam Lightning-Link conjugation kits (ab236553 and ab269820) following the manufacturer’s procedure. Slides were mounted using Vectashield with DAPI (Vector Laboratories). Representative images were taken using a Zeiss 980 Confocal Imaging System under ×63 magnification, and images of the whole slides were scanned using a Nikon ECLIPSE Ti2-E fluorescence microscope for quantification. Quantification of KRT5 and HER2 signal intensity from individual tumor cells was conducted using QuPath (35). Tumor cells were identified based on their location and nuclear morphology in the immunofluorescence image and were also confirmed on H&E-stained slides.

### RNA extraction and sequencing.

Total RNA was isolated from FFPE tissue specimens; each sample consisted of seven 4-μm sections scraped from individual slides. Paraffin wax was removed with 2 rounds of xylene extraction. Samples were incubated in lysis buffer containing proteinase K, and RNA was purified using miRNeasy FFPE (QIAGEN) kits following the manufacturer’s recommended procedure. RNA purity and concentration were measured by UV absorbance. RNA integrity (fragment size) was assessed using a 2100 Bioanalyzer and RNA 6000 Nano and 6000 Pico kits (Agilent Technologies). DV200 values (percentage of fragments with >200 bases) were calculated using 2100 Bioanalyzer Expert Software (Agilent Technologies). DV200 values ranged from 7% to 66% with 221 samples having a value ≥30%. Libraries were prepared using the Illumina RNA with Enrichment, Tagmentation protocol with the Illumina Exome Panel – Enrichment Oligos (Illumina). Libraries were profiled on a Bioanalyzer (Agilent) and quantified with an NGS Quantification Kit (Roche/Kapa Biosystems) using a StepOnePlus Real Time PCR Workstation (Thermo Fisher Scientific/Applied Biosystems International). Libraries were sequenced on a NovaSeq 6000 (Illumina) with RTA 3.4.4. All samples were randomized into different batches before sequencing to avoid potential confounding batch and biological effects. FASTQ files were assembled using bcl2fastq (Illumina).

### RNA-Seq processing and data analysis.

FASTQ files were trimmed of adapters using TrimGalore, then aligned to hg38 using STAR (v2.4.2a). FastQC was also run on each of the samples. Genes with 0 counts across all samples were filtered out. Differentially expressed genes were identified using DESeq2 (36) with raw counts as input. We set cutoff of *P*_adj_ < 0.05 and |FC| > 8 for pre-to-posttreatment biopsy comparisons and padj < 0.05 and |FC| > 1.5 for pretreatment sample comparisons. For the rest of analysis, log_2_-transformed trimmed mean of M values–normalized (TMM-normalized) counts per million (CPM) [log_2_(TMM-CPM + 1)] was used, in which the gene-level counts from all studies were normalized using TMM with edgeR (37). Principal component analysis (PCA) was performed using the edgeR package with PC1 and PC2 computed by the prcomp function. Seven samples were excluded from the downstream analysis because the PC2 distribution (treatment-unrelated component) was located more than 5 times the standard deviation apart from the mean of all samples. Further investigation showed that 6 of 7 samples showed aberrantly higher noncoding RNA expression and 1 sample showed extremely low expression of protein-coding genes, suggesting potential technical artifact. We also determined that adding these samples back did not affect any of the major conclusions of this study. For PI3K/ERBB2 gene signature enrichment scores and unbiased pathway alteration analysis, the GSVA package was used (38). Particularly, Reactome, KEGG, or BioCarta gene set collections were first scored in all the individual samples. Delta GSVA scores were then calculated by subtracting from the corresponding control groups (e.g., non-HET/pCR tumors or pretreatment tumors). The top 10 consistently upregulated or downregulated pathways or uniquely altered pathways from each collection were then plotted in the heatmaps. PAM50 subtype prediction was performed using the genefu package, and computed subtype probabilities were used to infer the likelihood of each subtype in each sample. Tumor overall immune and stromal scores were predicted using the ESTIMATE package (20) based on normalized gene expression in log_2_(CPM + 1) format. Illumina mode was selected for the platform option. Immune cell subtype abundance was predicted using CIBERSORT (39). *PIK3CA* and *ERBB2* hotspot mutations were screened by visualization of the BAM file in the Integrative Genomics Viewer browser at *PIK3CA* (exons 5, 8, 10, and 21) and *ERBB2* (exons 11, 22, and 23) regions. Samples without reads covering the hotspot regions were excluded for downstream analysis. TCR and BCR repertoires were inferred using the TRUST4 algorithm (22) by extraction of CDR3 region reads from BAM files. Clonotype richness and 4 diversity scores were calculated using the immunarch package (40).

### NanoString digital spatial profiling.

DSP analysis of surface antigens was performed by NanoString spatial microscopy on FFPE pretreatment tumor biopsies. Slides were stained with 61 oligo-conjugated antibodies. PanCK was used as a visualization marker to identify tumor-rich regions of interest (ROIs) and CD45/CD3 to identify immune-infiltrated ROIs. Per sample, 6 ROIs were selected within the tumor area, 3 with low immune cell infiltration and 3 with high immune cell infiltration. After hybridization of probes to slide-mounted FFPE tissue sections, the oligonucleotide tags were released from the tissue ROIs via UV radiation exposure. Released tags were quantitated in a standard nCounter assay (NanoString Technologies).

### Patients.

The details of the clinical trial have been previously reported (5). Briefly, a total of 164 patients were enrolled in the phase II neoadjuvant clinical trial of T-DM1 and pertuzumab (NCT02326974) from January 2015 to January 2018. The clinical trial was open at the Dana-Farber Cancer Institute, Massachusetts General Hospital, Sarah Cannon Research Institute, Tennessee Oncology, and Vanderbilt–Ingram Cancer Center. Patient enrollment required a pathologic diagnosis of carcinoma of the breast with HER2 IHC staining score of 3+ or *ERBB2* amplification based on FISH (ratio of *ERBB2* to chromosome 17 centromeric probe [CEP17] ≥ 2 or *ERBB2* copy number ≥ 6). HER2 status was centrally confirmed before study enrollment. The invasive tumor had to measure at least 2 cm in the greatest dimension assessed by physical examination or imaging; there was no upper limit on tumor size or axillary nodal status. Other requirements included willingness to undergo a research biopsy prior to treatment initiation, adequate hematopoietic and liver function, and a left ventricular ejection fraction of 55% or greater. Patients received 6 cycles of T-DM1 and pertuzumab (Figure 1A).

Of all patients, 163 were treated with at least 1 dose of T-DM1 and pertuzumab. Treatment consisted of 6 cycles of T-DM1 given in combination with pertuzumab. Participants received T-DM1 at a dose of 3.6 mg per kilogram of body weight, and pertuzumab at a loading dose of 840 mg followed by 420 mg. T-DM1 and pertuzumab were given every 3 weeks intravenously for a total duration of 6 cycles. Patients underwent breast surgery within 42 days of the last cycle of therapy. The type of breast surgery was at the discretion of the patient and surgeon. Decisions regarding choices of adjuvant radiotherapy and adjuvant systemic therapy were made by the treatment team and not mandated per protocol. Central confirmation of HER2 status to define eligibility classified 74% (121/163) of cases as HER2 3+ by IHC and 25% (40/163) as HER2 2+. HER2 2+ cases were confirmed to be HER2-positive by FISH prior to study enrollment using FFPE material from diagnostic biopsies. HER2 positivity was defined by FISH without IHC information in 2 cases (1%, 2/163). All but 1 patient (99.4%) had either stage II or III cancer at presentation. Two-thirds (68.7%) of tumors were classified as hormone receptor–positive (HR-positive) and the remaining tumors as HR-negative.

Pathologic response was reported using the RCB calculator. RCB 0, no residual invasive cancer in the breast or axillary nodes, defined pCR for the primary endpoint of the study. Left ventricular ejection fraction was assessed at baseline, end of cycle 2, and the presurgery visit. Laboratory monitoring was performed prior to each treatment cycle, and adverse events were assessed with each treatment cycle according to Common Terminology Criteria for Adverse Events v4.0. Single-cell HER2 counting by FISH was used to evaluate HER2 heterogeneity as a continuous variable. The fraction of HER2-non-amplified cells was evaluated in 3 areas per core biopsy site at the central pathology laboratory in Milan. The protocol was reviewed by the Dana-Farber/Harvard Cancer Center Data and Safety Monitoring Committee throughout the study to monitor toxicity and review accrual.

Our translational research analyses were performed using pCR and no pCR status defined as RCB-0 and non–RCB-0 in most of the comparisons or RCB scores evaluated as a continuous variable. Image-guided research biopsies performed prior to treatment initiation were used for central pathology evaluation of HER2 heterogeneity and molecular profiling studies. Pathology material from residual tumors (i.e., no pCR) was used for central pathology evaluation and molecular profiling. A total of 292 research biopsies from 129 of these patients were used for transcriptomic profiling.

### Statistics.

All data were analyzed and plotted using R version 4.3.1 and GraphPad Prism 9.0 software. Box plots span the upper quartile (upper limit), median (center), and lower quartile (lower limit). Whiskers extend a maximum of 1.5 × IQR. Each individual data point is shown. All the comparisons were conducted at the patient level, where the mean measurement values of the 2 pretreatment biopsies were calculated for either intrapretreatment group or pre-to-posttreatment group comparisons, in order to ensure the independence of all the observations in each group. Pairwise and 2-tailed Mann-Whitney *U* test (unpaired samples) or Wilcoxon’s matched-pairs signed-rank test (paired samples) was used to compare the medians of each group. Pearson’s correlation was applied to derive the *P* value of correlative analysis. *P* values less than 0.05 were considered statistically significant.

### Study approval.

The institutional review board at each participating institution approved the study (Dana-Farber Cancer Institute IRB#14-409). Written informed consent was provided by all participants.

### Data availability.

RNA-Seq data were deposited to the NCBI’s Gene Expression Omnibus database under accession number GSE243375. A Supporting Data Values file with all reported data values is available as part of the supplemental material.

## Author contributions

ZL was responsible for data curation, formal analysis, investigation, methodology, writing of the original draft, and review and editing. OMF was responsible for conceptualization, resources, funding acquisition, and review and editing. GV was responsible for formal analysis, investigation, methodology, and supervision. PDO was responsible for formal analysis, investigation, and methodology. LR was responsible for formal analysis, investigation, and methodology. MAG was responsible for formal analysis, methodology, investigation, and review and editing. AK participated in the investigation. DAY was responsible for resources, data curation, and review and editing. VGA was responsible for resources, data curation, and review and editing. CLA was responsible for resources, data curation, and review and editing. LMS was responsible for resources, data curation, and review and editing. KC was responsible for formal analysis, methodology, and investigation. CH was responsible for formal analysis, methodology, and investigation. AGW was responsible for resources, data curation, and review and editing. TAK was responsible for resources, data curation, and review and editing. SL was responsible for resources, data curation, and review and editing. JRB was responsible for resources, data curation, and review and editing. EPW was responsible for conceptualization, resources, funding acquisition, and review and editing. PTS was responsible for resources, funding acquisition, supervision, and review and editing. IEK was responsible for conceptualization, resources, funding acquisition, and review and editing. KP was responsible for conceptualization, resources, supervision, funding acquisition, writing of the original draft, and review and editing.

## Supplementary Material

Supplemental data

ICMJE disclosure forms

Supplemental table 1

Supplemental table 2

Supplemental table 3

Supporting data values

## Version 1 02/01/2024

In-Press Preview

## Version 2 04/01/2024

Electronic publication
