# Supplementary material for: HER2 heterogeneity and treatment response–associated profiles in HER2-positive breast cancer in the NCT02326974 clinical trial
Source: J Clin Invest. 2024 Feb 1;134(7):e176454. doi: 10.1172/JCI176454 (PMC10977978; doi:10.1172/JCI176454)
Supplement: Supplemental data [file jci-134-176454-s201.pdf]

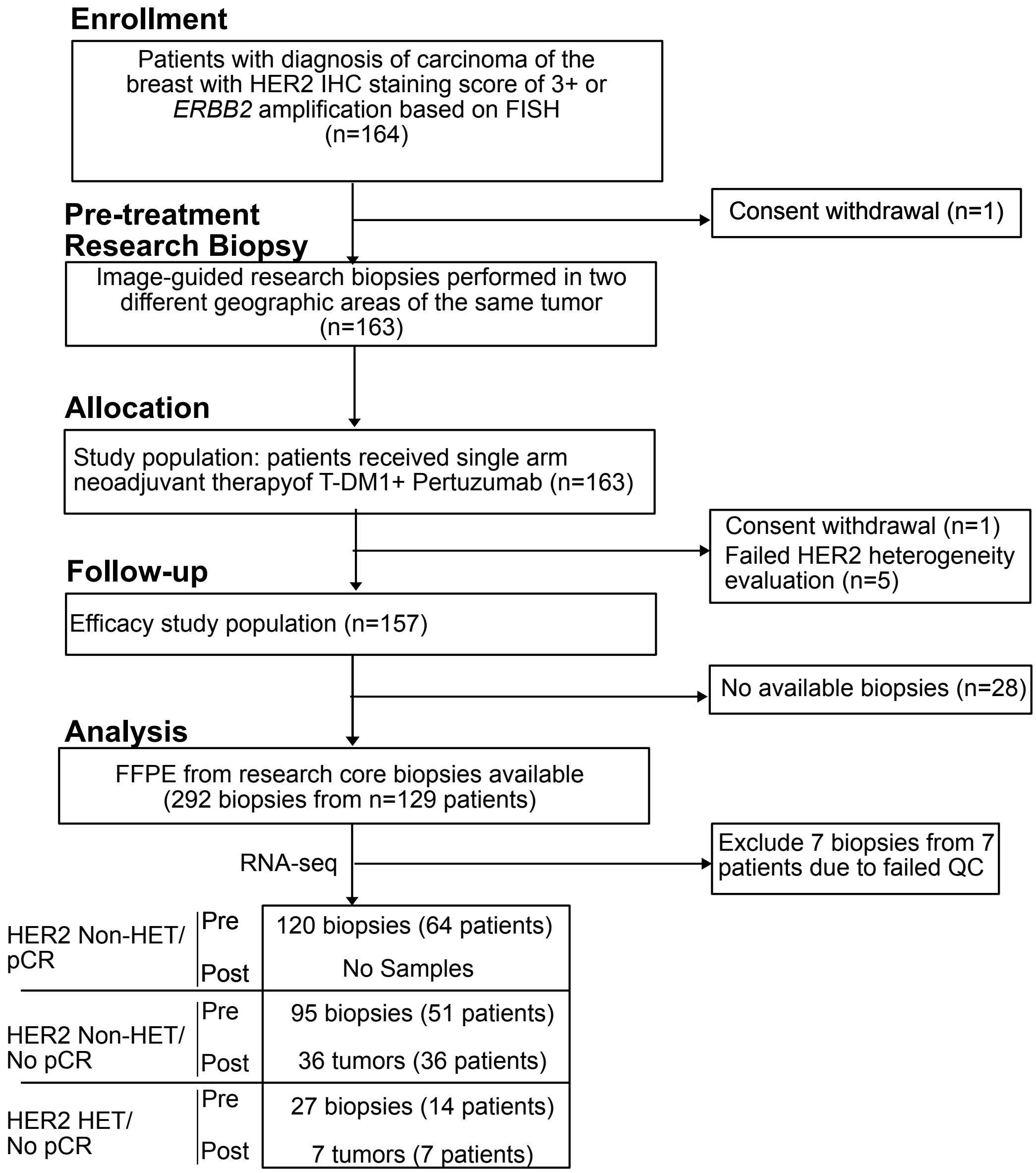

Enrollment
Patients with diagnosis of carcinoma of the breast with HER2 IHC staining score of 3+ or ERBB2 amplification based on FISH (n=164)
Consent withdrawal (n=1)
Pre-treatment Research Biopsy
Image-guided research biopsies performed in two different geographic areas of the same tumor (n=163)
Allocation
Study population: patients received single arm neoadjuvant therapyof T-DM1+ Pertuzumab (n=163)
Consent withdrawal (n=1)
Failed HER2 heterogeneity evaluation (n=5)
Follow-up
Efficacy study population (n=157)
No available biopsies (n=28)
Analysis
FFPE from research core biopsies available (292 biopsies from n=129 patients)
RNA-seq
Exclude 7 biopsies from 7 patients due to failed QC
HER2 Non-HET/ pCR
Pre
120 biopsies (64 patients)
Post
No Samples
HER2 Non-HET/ No pCR
Pre
95 biopsies (51 patients)
Post
36 tumors (36 patients)
HER2 HET/ No pCR
Pre
27 biopsies (14 patients)
Post
7 tumors (7 patients)

Suppl Fig. 1

Supplemental Figure 1. CONSORT diagram depicting study design. Flow chart summarizing the study enrollment criteria, patient allocation, follow-up information, and analyses performed. Patients or sample excluded at different stages are indicated. Numbers of biopsies and patients in the three clinically distinct subgroups are listed.

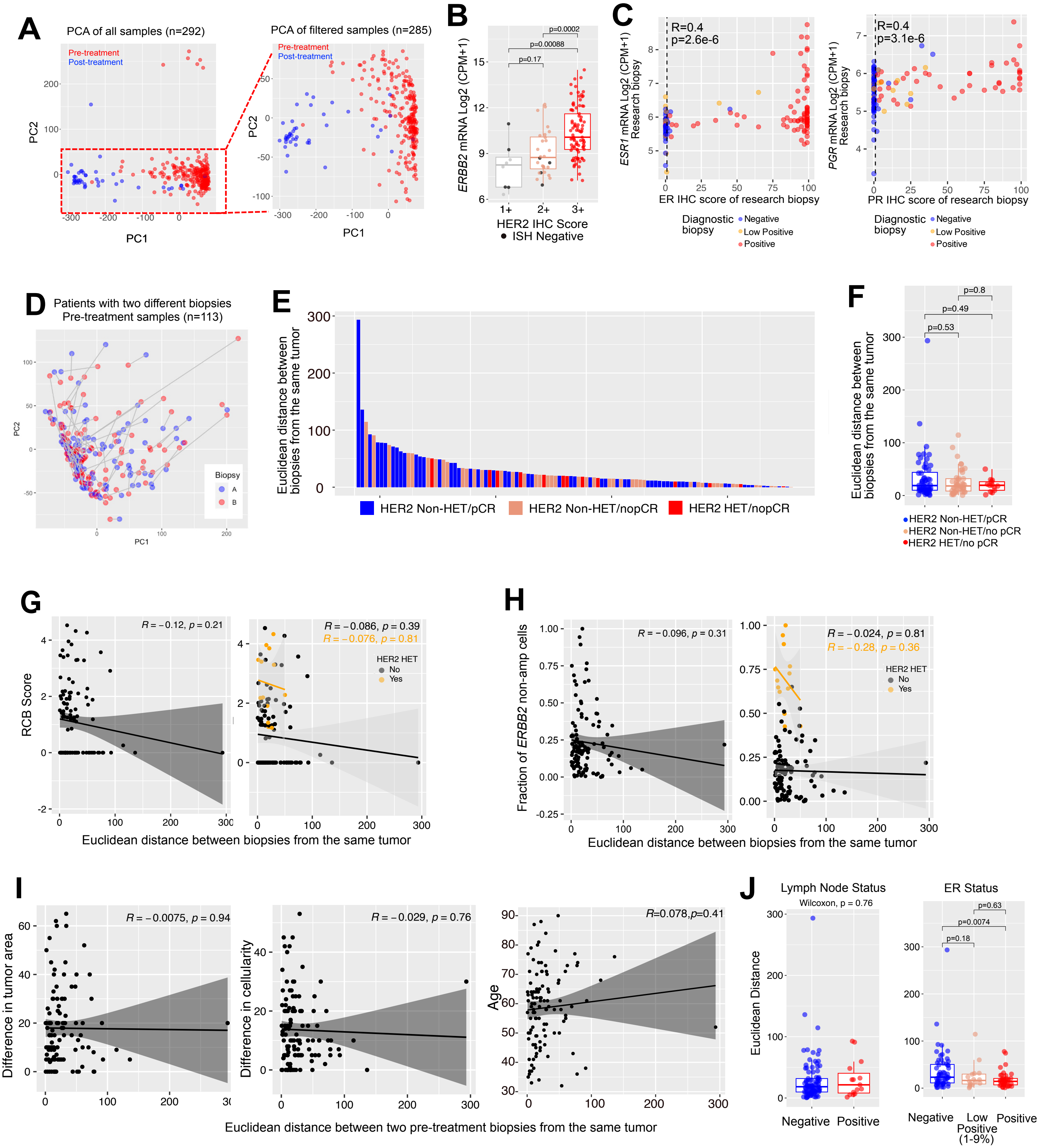
A
PCA of all samples (n=292)
Pre-treatment
Post-treatment
PC2
PC1
PCA of filtered samples (n=285)
B
ERBB2 mRNA Log2 (CPM+1)
p=0.0002
p=0.00088
p=0.17
HER2 IHC Score
ISH Negative
C
ESR1 mRNA Log2 (CPM+1) Research biopsy
R=0.4
p=2.6e-6
ER IHC score of research biopsy
PGR mRNA Log2 (CPM+1) Research biopsy
R=0.4
p=3.1e-6
PR IHC score of research biopsy
Diagnostic biopsy
Negative
Low Positive
Positive
D
Patients with two different biopsies
Pre-treatment samples (n=113)
Biopsy
A
B
E
Euclidean distance between biopsies from the same tumor
HER2 Non-HET/pCR
HER2 Non-HET/nopCR
HER2 HET/nopCR
F
p=0.8
p=0.49
p=0.53
HER2 Non-HET/pCR
HER2 Non-HET/no pCR
HER2 HET/no pCR
G
RCB Score
R = −0.12, p = 0.21
R = −0.086, p = 0.39
R = −0.076, p = 0.81
HER2 HET
No
Yes
Euclidean distance between biopsies from the same tumor
H
Fraction of ERBB2 non-amp cells
R = −0.096, p = 0.31
R = −0.024, p = 0.81
R = −0.28, p = 0.36
I
Difference in tumor area
R = −0.0075, p = 0.94
Difference in cellularity
R = −0.029, p = 0.76
Age
R=0.078,p=0.41
Euclidean distance between two pre-treatment biopsies from the same tumor
J
Lymph Node Status
Wilcoxon, p = 0.76
Euclidean Distance
Negative
Positive
ER Status
p=0.63
p=0.0074
p=0.18
Negative
Low Positive (1-9%)
Positive

Suppl Fig. 2

Supplemental Figure 2. Quality analyses of RNA-seq data and transcriptomic variation in intra-patient paired biopsies. (**A**) PCA plot showing transcriptomic variation in all 292 samples. Seven samples identified as outliers were removed from subsequent analyses, the remaining 285 samples were replotted in the right panel. (**B**) Box plot showing *ERBB2* mRNA expression from RNA-seq among patients with different HER2 scores based on immunohistochemistry (IHC). Mean expression of two biopsies from the same patient was used. *ERBB2* ISH (in situ hybridization) negative cases are marked in the plot. (**C**) Scatter plots depicting Pearson correlation between mRNA (RNA-seq) and protein (IHC) levels for *ESR1* and *PGR* in pre-treatment samples assessed in research biopsies. Mean measurement of ER and PR IHC scores and mRNA expression from two pre-treatment biopsies of the same patient was used for plotting. Colors represent ER and PR IHC results of the diagnostic biopsy of the corresponding patients. (**D**) PCA plot showing transcriptomic variation of 113 intra-patient paired pre-treatment biopsies. Two biopsies from the same patients are connected with gray line. (**E**) Histogram illustrating the distance of each biopsy pairs in (D) and their indicated groups based on pCR and HER2 heterogeneity. (**F**) Box plots comparing transcriptomic distances in paired pre-treatment biopsies. (**G,H**) Scatter plots showing Pearson correlation between transcriptomic distance of intra-patient biopsy pairs and RCB scores (**G**) and HER2 negative ratios accessed by FISH (**H**) in all samples (left) or within HER2 HET and Non-HET cases (right). (**I**) Scatter plots showing the Pearson correlation between transcriptomic distances (x-axis) and differences in tumor areas (y-axis, left), tumor cell cellularity (y-axis, middle) or age (y-axis, right) in intra-patient biopsy pairs. (**J**) Box plots depicting transcriptomic distances between intra-patient biopsy pairs in patients with different nodal status and ER positivity. P values were calculated based on two tailed Mann-Whitney U test for **B,F** and **J** and Pearson correlation for **C,G,H** and **I**.

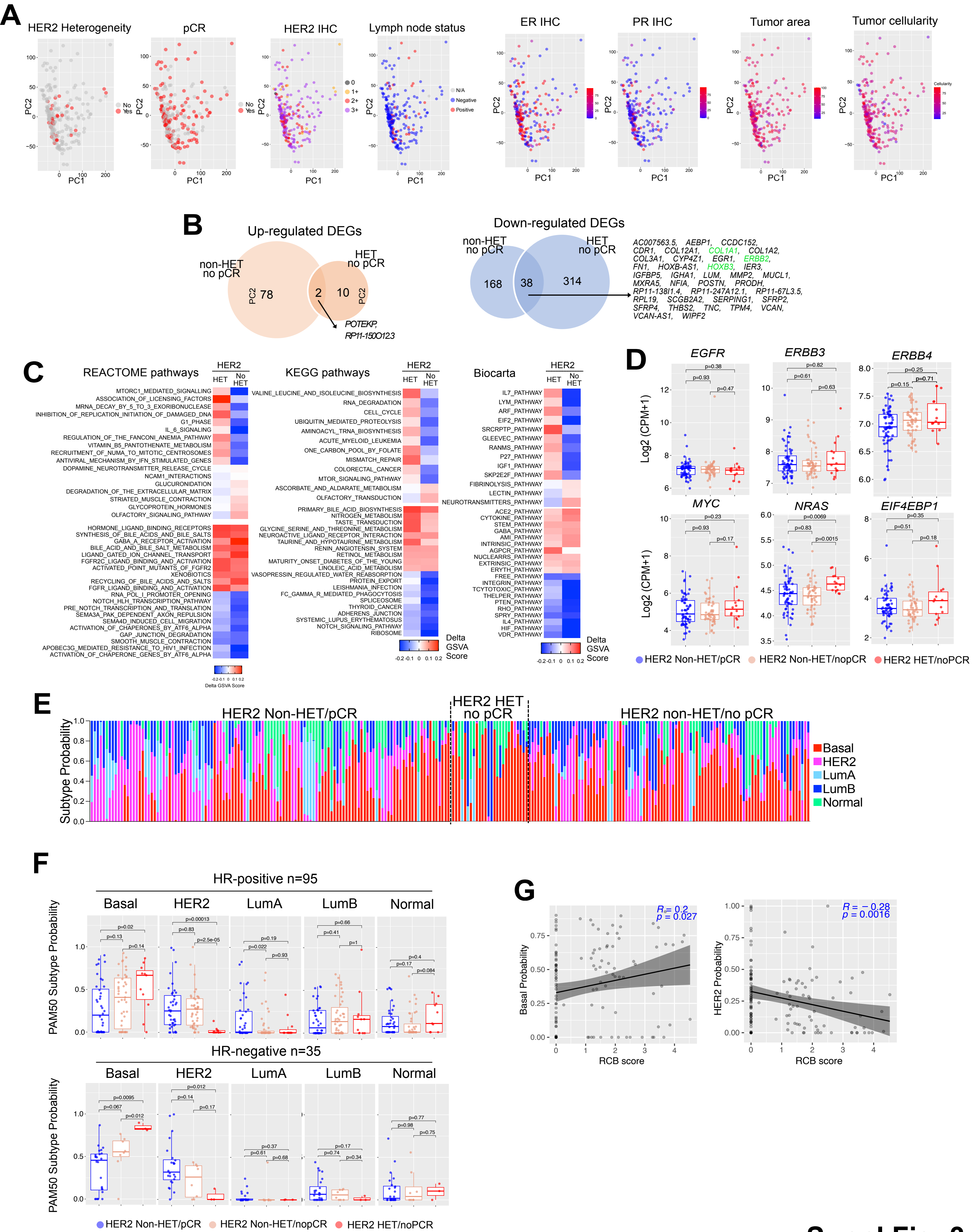
A
HER2 Heterogeneity
pCR
HER2 IHC
Lymph node status
ER IHC
PR IHC
Tumor area
Tumor cellularity
B
Up-regulated DEGs
non-HET no pCR
HET no pCR
78
2
10
POTEKP, RP11-150O12.3
Down-regulated DEGs
168
38
314
AC007563.5, AEBP1, CCDC152, CDR1, COL12A1, COL1A1, COL1A2, COL3A1, CYP4Z1, EGR1, ERBB2, FN1, HOXB-AS1, HOXB3, IER3, IGFBP5, IGHA1, LUM, MMP2, MUCL1, MXRA5, NFIA, POSTN, PRODH, RP11-138I1.4, RP11-247A12.1, RP11-67L3.5, RPL19, SCGB2A2, SERPING1, SFRP2, SFRP4, THBS2, TNC, TPM4, VCAN, VCAN-AS1, WIPF2
C
REACTOME pathways
KEGG pathways
Biocarta
Delta GSVA Score
D
EGFR
ERBB3
ERBB4
MYC
NRAS
EIF4EBP1
Log2 (CPM+1)
HER2 Non-HET/pCR
HER2 Non-HET/nopCR
HER2 HET/noPCR
E
HER2 Non-HET/pCR
HER2 HET no pCR
HER2 non-HET/no pCR
Subtype Probability
Basal
HER2
LumA
LumB
Normal
F
HR-positive n=95
HR-negative n=35
PAM50 Subtype Probability
G
R = 0.2
p = 0.027
R = − 0.28
p = 0.0016
Basal Probability
HER2 Probability
RCB score

Suppl Fig. 3

Supplemental Figure 3. HER2 heterogeneity and treatment-associated differences in gene expression patterns. (A) PCA plot depicting the distribution of 242 pretreatment samples separated by the indicated categorical and quantitative parameters. **(B)** Venn diagrams showing the overlap of DEGs between the indicated comparisons. Overlapping genes are listed. **(C)** Heatmaps showing top pathways differentially enriched in HER2 HET and Non-HET tumors normalized to pCR samples. Red and blue represents increased and decreased pathway enrichment compared to pCR samples. Color scale indicates magnitude of change. **(D)** Box plots showing expression level of ERBB gene family members and selected ERBB/PI3K pathway genes across the indicated groups using the mean of two biopsies from the same patient. **(F)** Stacked bar plot illustrating the PAM50 subtype probability among HER2 Non-HET/pCR, Non-HET/nopCR, and HET/nopCR samples. **(F)** Box plots depicting PAM50 subtype probability scores in the indicated groups separated by hormone receptor status using the mean score of two biopsies from the same patient. (**G**) Scatter plots showing the correlation between basal and HER2 subtype probability scores and RCB scores across all samples. P values were calculated based on two tailed Mann-Whitney U test in **D,F**, and Pearson correlation in **G**.

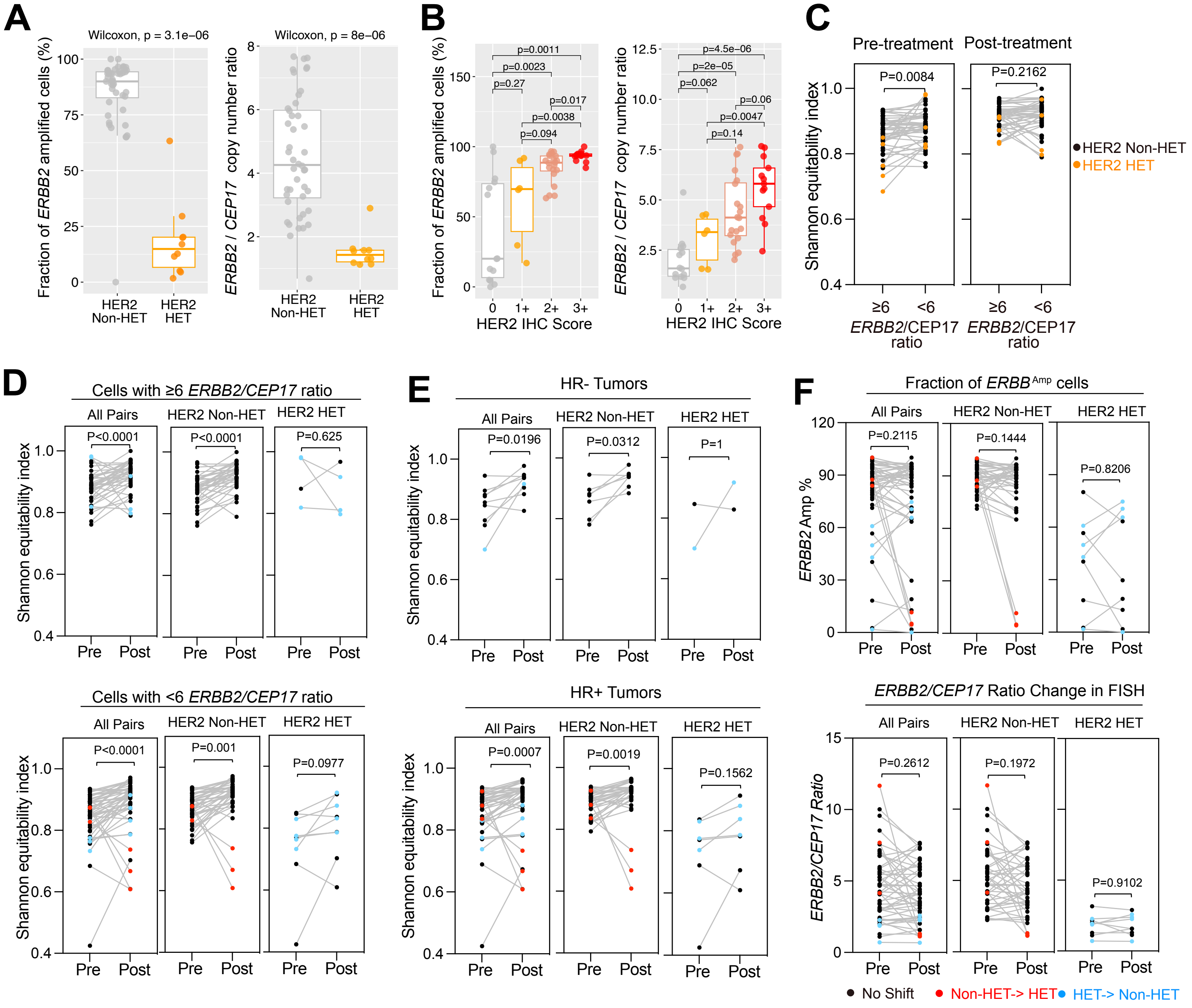

A
Wilcoxon, p = 3.1e−06
Fraction of ERBB2 amplified cells (%)
HER2 Non-HET
HER2 HET
Wilcoxon, p = 8e−06
ERBB2 / CEP17 copy number ratio
B
Fraction of ERBB2 amplified cells (%)
HER2 IHC Score
ERBB2 / CEP17 copy number ratio
C
Pre-treatment
Post-treatment
Shannon equitability index
P=0.0084
P=0.2162
ERBB2/CEP17 ratio
HER2 Non-HET
HER2 HET
D
Cells with ≥6 ERBB2/CEP17 ratio
All Pairs
HER2 Non-HET
HER2 HET
P<0.0001
P<0.0001
P=0.625
Pre
Post
Cells with <6 ERBB2/CEP17 ratio
P<0.0001
P=0.001
P=0.0977
E
HR- Tumors
P=0.0196
P=0.0312
P=1
HR+ Tumors
P=0.0007
P=0.0019
P=0.1562
F
Fraction of ERBB^Amp cells
P=0.2115
P=0.1444
P=0.8206
ERBB2 Amp %
ERBB2/CEP17 Ratio Change in FISH
P=0.2612
P=0.1972
P=0.9102
ERBB2/CEP17 Ratio
No Shift
Non-HET-> HET
HET-> Non-HET

Suppl. Fig. 4

Supplemental Figure 4. Treatment-induced HER2 heterogeneity alterations. (A)(B) Box plots showing the relative proportion of *ERBB2* amplified cells and ERBB2/CEP17 ratios in residual tumors classified as HER2 HET and Non-HET based on pretreatment biopsies (**A**) and with different HER2 IHC scores (**B**). **(C)** Line plots depicting the Shannon's equitability index between tumor cell populations with the indicated *ERBB2/CEP17* ratios within the same tumor (connected by line) in pre- (left) and post-treatment (right) samples. **D-(F)** Line plots showing changes in Shannon's equitability index (**(D)E**) the relative fraction of *ERBB2* amplified cells and in *ERBB2*/CEP17 ratios (**F**) in paired pre- and post-treatment biopsies among all tumors or within samples classified as HER2 Non-HET and HER2 HET based on FISH on pre-treatment biopsies. Samples with a change in HER2 heterogeneity are highlighted in red and blue. Two-tailed Mann Whitney U test was used in **(A) (B)** and Wilcoxon matched-pairs signed rank test was used in **(C) (D) E** and **F**.

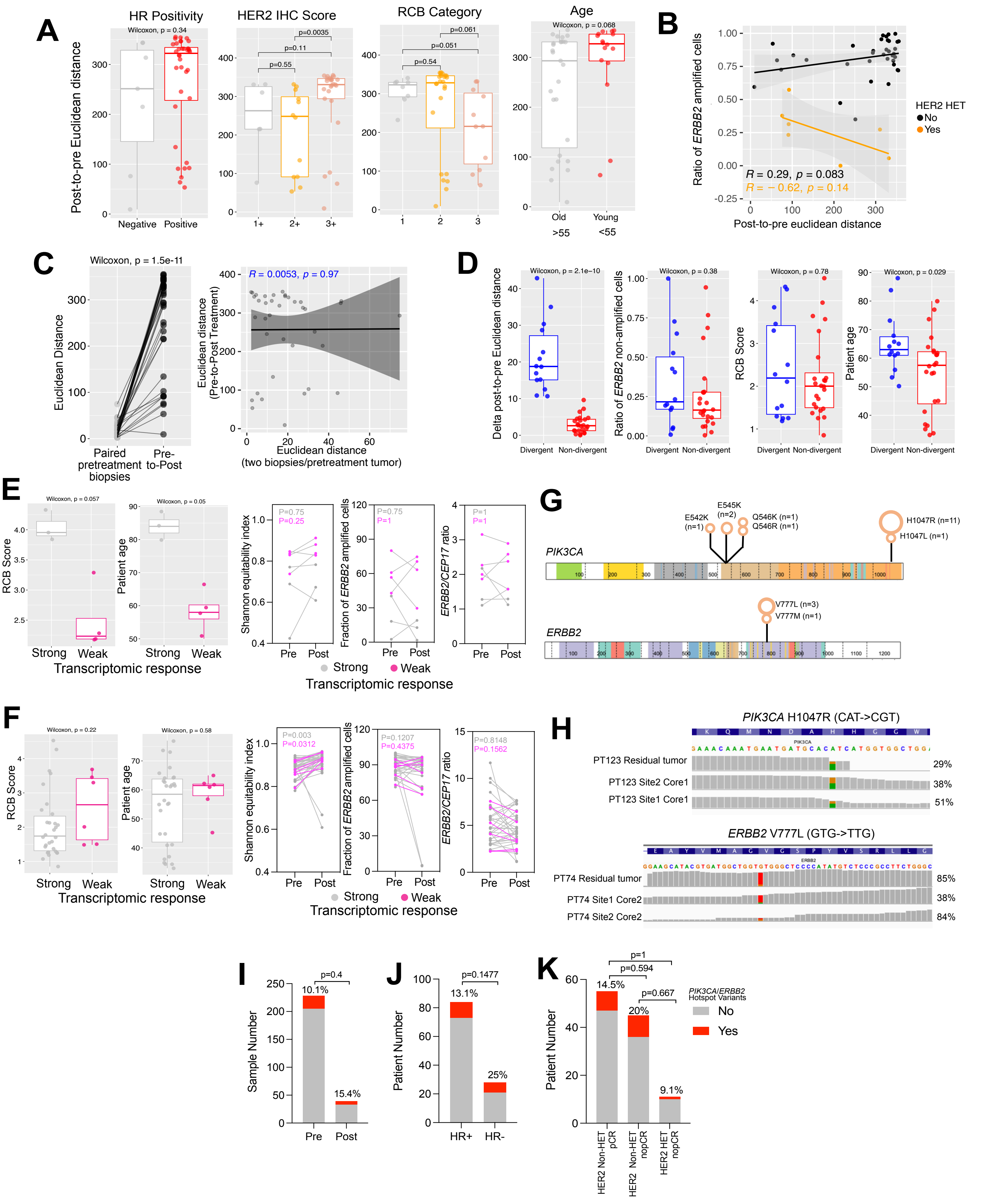

A
HR Positivity
HER2 IHC Score
RCB Category
Age
Post-to-pre Euclidean distance
Negative
Positive
Old >55
Young <55
B
Ratio of ERBB2 amplified cells
Post-to-pre euclidean distance
HER2 HET
No
Yes
R = 0.29, p = 0.083
R = − 0.62, p = 0.14
C
Euclidean Distance
Paired pretreatment biopsies
Pre-to-Post
Euclidean distance (Pre-to-Post Treatment)
Euclidean distance (two biopsies/pretreatment tumor)
R = 0.0053, p = 0.97
D
Delta post-to-pre Euclidean distance
Ratio of ERBB2 non-amplified cells
RCB Score
Patient age
Divergent
Non-divergent
E
Transcriptomic response
Strong
Weak
Shannon equitability index
Fraction of ERBB2 amplified cells
ERBB2/CEP17 ratio
Pre
Post
F
G
PIK3CA
ERBB2
E542K (n=1)
E545K (n=2)
Q546K (n=1)
Q546R (n=1)
H1047R (n=11)
H1047L (n=1)
V777L (n=3)
V777M (n=1)
H
PIK3CA H1047R (CAT->CGT)
PT123 Residual tumor 29%
PT123 Site2 Core1 38%
PT123 Site1 Core1 51%
ERBB2 V777L (GTG->TTG)
PT74 Residual tumor 85%
PT74 Site1 Core2 38%
PT74 Site2 Core2 84%
I
Sample Number
10.1%
15.4%
p=0.4
J
Patient Number
HR+
HR-
13.1%
25%
p=0.1477
K
14.5%
20%
9.1%
p=1
p=0.594
p=0.667
HER2 Non-HET pCR
HER2 Non-HET nopCR
HER2 HET nopCR
PIK3CA/ERBB2 Hotspot Variants

Suppl Fig 5

Supplemental Figure 5. Treatment-induced differences in transcriptomic profiles. (A) Box plots showing transcriptomic distances between pre- and post-treatment samples in the indicated patient groups, using the mean distance of two pre-treatment biopsies from the same patient for pre-treatment value. **(B)** Scatter plot illustrating Pearson correlation between transcriptomic distances between pre- and post-treatment samples and fraction of *ERBB2*^{amp} cells in HET and non-HET cases. Values represent the mean of two biopsies from the same patient. **(C)** Line plot showing Euclidean distances between two pre-treatment biopsies from the same tumor and the corresponding pre-to-post treatment samples from the same patient (left panel). Scatter plot depicting the correlation of these two distances (right panel). **(D)** Box plots depicting differences in treatment-induced Euclidean distance between the two intra-patient biopsies, and differences in *ERBB2* amplified cell ratio, RCB scores, and patient age in biopsies with divergent or non-divergent responses. **(E,F)** Box plots (left) showing the comparisons of RCB score and patient age between strong and weak transcriptomic responders and line plots (right) showing changes of Shannon's equitability index, fraction of *ERBB2* amplified cells and *ERBB2*/CEP17 copy number ratios in paired pre- and post-treatment samples classified as strong and weak transcriptomic responders in HER2 HET (**E**) and Non-HET (**F**) tumors. **(G)** Schematic illustration of hotspot mutations detected in *PIK3CA* and *ERBB2*. **(H)** Screenshot of representative cases with *PIK3CA* H1047R and *ERBB2* V777L mutations across all the biopsies. **(I-K)** Stacked bar plots showing enrichment of *PIK3CA* and *ERBB2* mutations in the indicated subgroups. P values were calculated based on two tailed Mann-Whitney U test in **A,D** and left panels of **E,F**, two-tailed Wilcoxon matched-pairs signed rank test in the left panel of **C** and the right panels of **E** and **F**, Pearson correlation in the right panel of **B,C** and Two-tailed Fisher's Exact test in **I, J** and **K**.

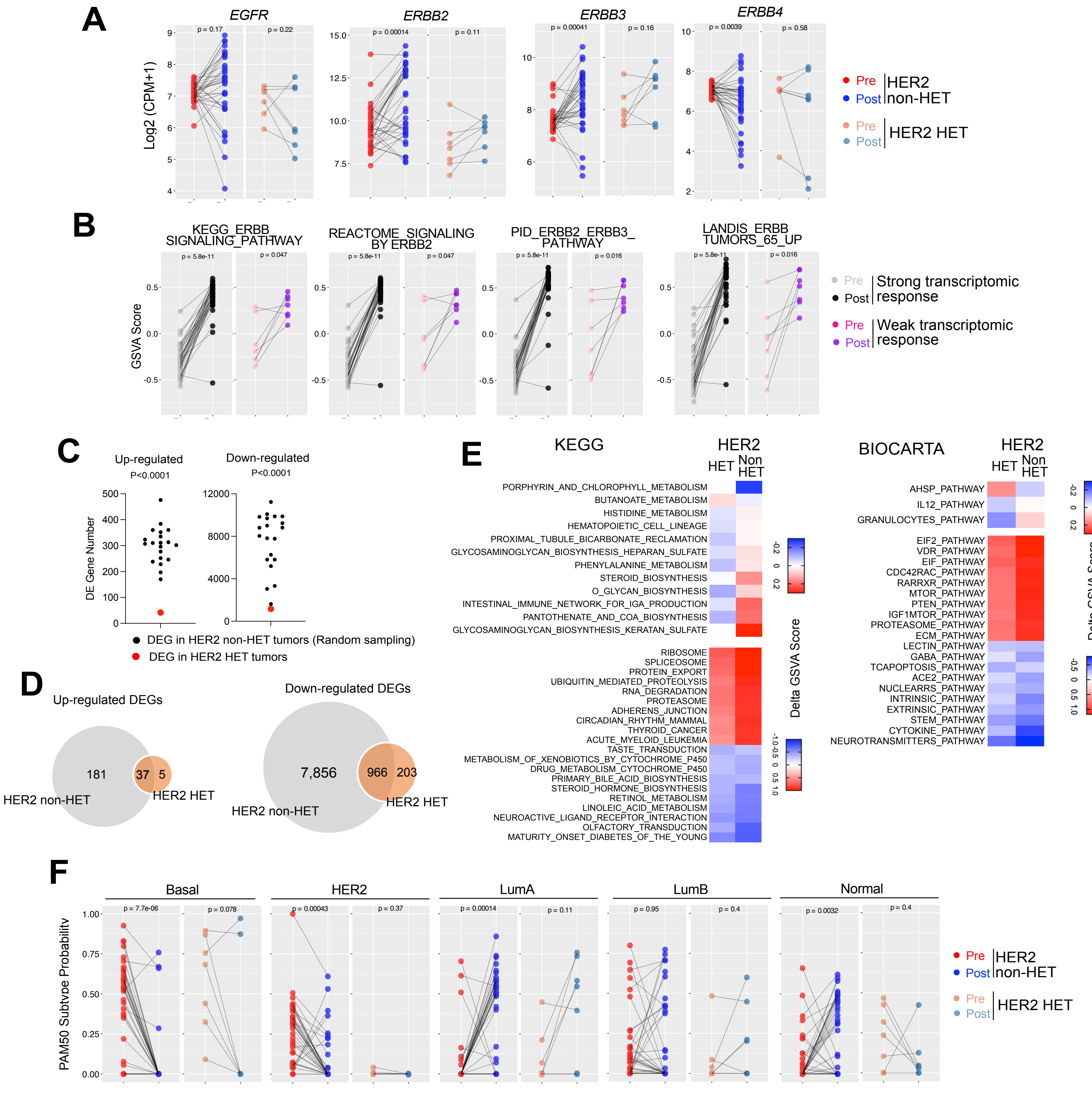

A
EGFR
ERBB2
ERBB3
ERBB4
Log2 (CPM+1)
p = 0.17
p = 0.22
p = 0.00014
p = 0.11
p = 0.00041
p = 0.16
p = 0.0039
p = 0.58
Pre
Post
HER2 non-HET
HER2 HET
B
KEGG_ERBB SIGNALING_PATHWAY
REACTOME_SIGNALING BY ERBB2
PID_ERBB2_ERBB3_PATHWAY
LANDIS_ERBB TUMORS_65_UP
GSVA Score
p = 5.8e-11
p = 0.047
p = 0.016
Strong transcriptomic response
Weak transcriptomic response
C
Up-regulated
Down-regulated
P<0.0001
DE Gene Number
DEG in HER2 non-HET tumors (Random sampling)
DEG in HER2 HET tumors
D
Up-regulated DEGs
Down-regulated DEGs
181
37
5
7,856
966
203
HER2 non-HET
HER2 HET
E
KEGG
HER2
HET
Non HET
PORPHYRIN_AND_CHLOROPHYLL_METABOLISM
BUTANOATE_METABOLISM
HISTIDINE_METABOLISM
HEMATOPOIETIC_CELL_LINEAGE
PROXIMAL_TUBULE_BICARBONATE_RECLAMATION
GLYCOSAMINOGLYCAN_BIOSYNTHESIS_HEPARAN_SULFATE
PHENYLALANINE_METABOLISM
STEROID_BIOSYNTHESIS
O_GLYCAN_BIOSYNTHESIS
INTESTINAL_IMMUNE_NETWORK_FOR_IGA_PRODUCTION
PANTOTHENATE_AND_COA_BIOSYNTHESIS
GLYCOSAMINOGLYCAN_BIOSYNTHESIS_KERATAN_SULFATE
RIBOSOME
SPLICEOSOME
PROTEIN_EXPORT
UBIQUITIN_MEDIATED_PROTEOLYSIS
RNA_DEGRADATION
PROTEASOME
ADHERENS_JUNCTION
CIRCADIAN_RHYTHM_MAMMAL
THYROID_CANCER
ACUTE_MYELOID_LEUKEMIA
TASTE_TRANSDUCTION
METABOLISM_OF_XENOBIOTICS_BY_CYTOCHROME_P450
DRUG_METABOLISM_CYTOCHROME_P450
PRIMARY_BILE_ACID_BIOSYNTHESIS
STEROID_HORMONE_BIOSYNTHESIS
RETINOL_METABOLISM
LINOLEIC_ACID_METABOLISM
NEUROACTIVE_LIGAND_RECEPTOR_INTERACTION
OLFACTORY_TRANSDUCTION
MATURITY_ONSET_DIABETES_OF_THE_YOUNG
Delta GSVA Score
BIOCARTA
AHSP_PATHWAY
IL12_PATHWAY
GRANULOCYTES_PATHWAY
EIF2_PATHWAY
VDR_PATHWAY
EIF_PATHWAY
CDC42RAC_PATHWAY
RARRXR_PATHWAY
MTOR_PATHWAY
PTEN_PATHWAY
IGF1MTOR_PATHWAY
PROTEASOME_PATHWAY
ECM_PATHWAY
LECTIN_PATHWAY
GABA_PATHWAY
TCAPOPTOSIS_PATHWAY
ACE2_PATHWAY
NUCLEARRS_PATHWAY
INTRINSIC_PATHWAY
EXTRINSIC_PATHWAY
STEM_PATHWAY
CYTOKINE_PATHWAY
NEUROTRANSMITTERS_PATHWAY
F
Basal
HER2
LumA
LumB
Normal
PAM50 Subtype Probability
p = 7.7e-06
p = 0.078
p = 0.00043
p = 0.37
p = 0.00014
p = 0.11
p = 0.95
p = 0.4
p = 0.0032
p = 0.4

Suppl Fig. 6

Supplemental Figure 6. Treatment-induced transcriptomic changes. (A,B) Line plots showing the expression of ERBB gene family members in pre- and post-treatment tumors subdivided based on HER2 heterogeneity (**A**), and enrichments scores of the four ERBB signaling signatures in the pre- and post-treatment tumors divided by transcriptomic response type (**B**). Average measurements from the two pre-treatment biopsies of the same patient were used for the comparisons. (**C**) Dot plots depicting numbers of DEGs with higher or lower expression levels in post- compared to pre-treatment samples. Red and black dots represent DEGs in HER2 heterogenous and non-heterogeneous group, respectively, with the latter one taking 20 random sampling of the same numbers as HER2 heterogenous group to correct for differences in sample size. **(D)** Venn diagrams showing the overlap of DEGs up- and downregulated between post and pretreatment samples within HER2 heterogeneous and non-heterogeneous groups. **(E)** Heatmaps of top KEGG and BIOCARTA pathways differentially enriched between pre- and post-treatment samples from HER2 Non-HET and HET cases. Red and blue represents increased and decreased pathway enrichment in post-treatment samples compared to pre-treatment counterparts. Color scale indicates magnitude of change. (**F**) Line plots depicting PAM50 subtype probability scores in pre- and post-treatment tumors with the indicated HER2 heterogeneity status at patient level where the average scores from the two pre-treatment biopsies of the same patient were used for pre-treatment values. P values were calculated based on two-tailed Wilcoxon matched-pairs signed rank test in **A,B,F** and two-tailed one sample t test in **C**.

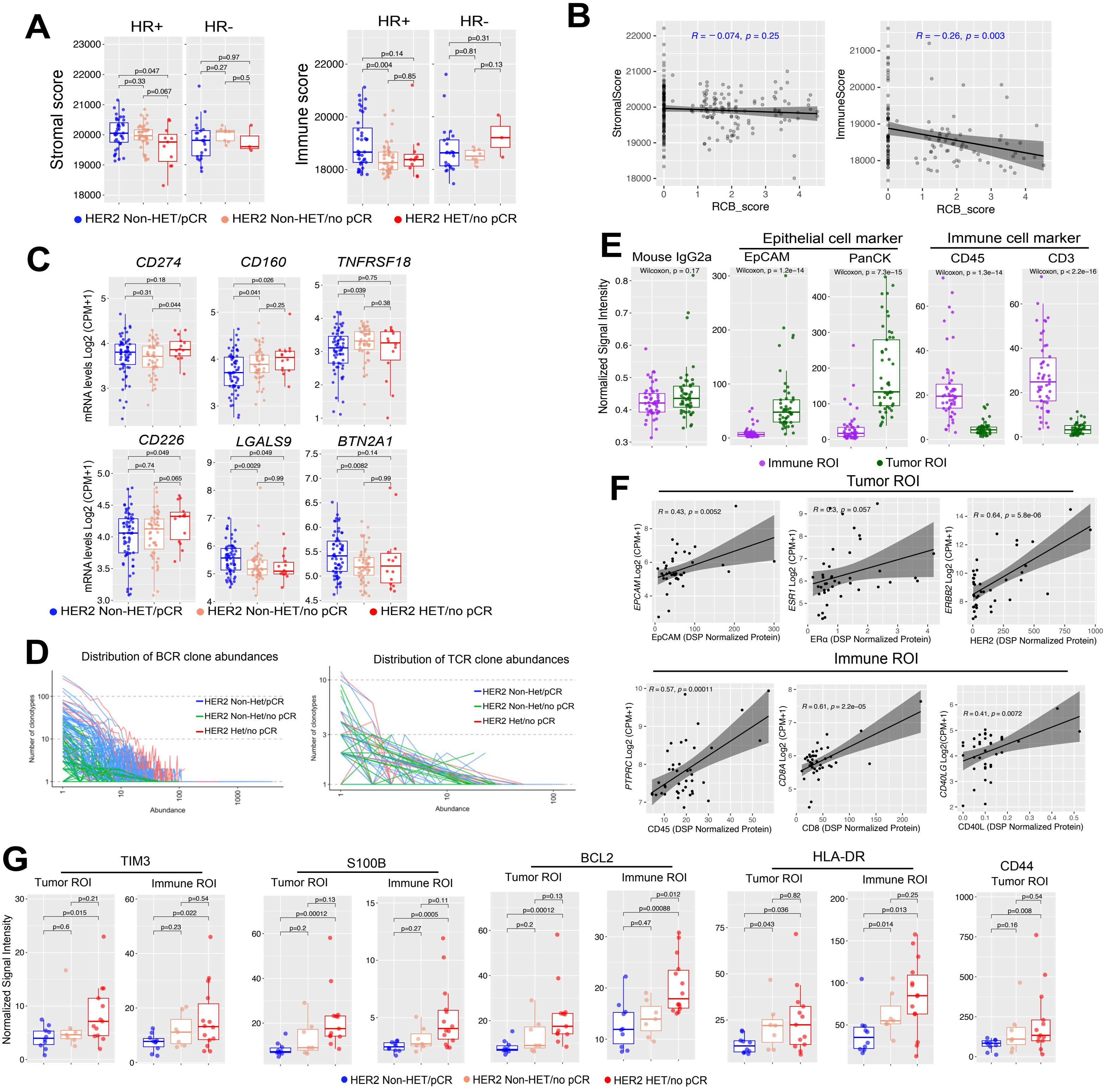

A
HR+
HR-
Stromal score
Immune score
HER2 Non-HET/pCR
HER2 Non-HET/no pCR
HER2 HET/no pCR
B
StromalScore
ImmuneScore
RCB_score
R = −0.074, p = 0.25
R = −0.26, p = 0.003
C
CD274
CD160
TNFRSF18
CD226
LGALS9
BTN2A1
mRNA levels Log2 (CPM+1)
D
Distribution of BCR clone abundances
Distribution of TCR clone abundances
Number of clonotypes
Abundance
HER2 Non-Het/pCR
HER2 Non-Het/no pCR
HER2 Het/no pCR
E
Epithelial cell marker
Immune cell marker
Mouse IgG2a
EpCAM
PanCK
CD45
CD3
Normalized Signal Intensity
Immune ROI
Tumor ROI
F
Tumor ROI
Immune ROI
EpCAM (DSP Normalized Protein)
ERα (DSP Normalized Protein)
HER2 (DSP Normalized Protein)
CD45 (DSP Normalized Protein)
CD8 (DSP Normalized Protein)
CD40L (DSP Normalized Protein)
G
TIM3
S100B
BCL2
HLA-DR
CD44
Tumor ROI
Immune ROI
Normalized Signal Intensity

Suppl Fig. 7

Supplemental Figure 7. HER2 heterogeneity-associated differences in the immune microenvironment in pretreatment samples. (A) Box plots depicting predicted overall stromal and immune scores in pre-treatment tumors within the indicated patient groups subgrouped by hormone receptor (HR) status. Mean scores from the two pre-treatment biopsies from the same patient were used. **(B)** Scatter plot showing the correlation of stromal and immune scores with RCB scores at patient level. **(C)** Box plots showing expression level of genes encoding immune checkpoint proteins among the indicated groups, where mean expression value from the two pre-treatment biopsies were calculated for pre-trearment value. **(D)** Line plots showing distribution of BCR (left panel) and TCR (right panel) clonotype abundances of each individual pre-treatment sample. **(E)** Box plots depicting the comparison DSP signal intensities of negative control, tumor and immune markers in tumor and immune ROIs in all the samples. **(F)** Scatter plots showing the Pearson correlation between mRNA (RNA-seq) and protein expression (DSP immune or tumor ROIs) of six targets. **(G)** Box plots showing the normalized expression of indicated targets from DSP among the three groups in pre-treatment samples. Tumor or immune ROI was labelled. P values were calculated based on two tailed Mann-Whitney U test for **A,C,E,G** and Pearson correlation in **B,F**.

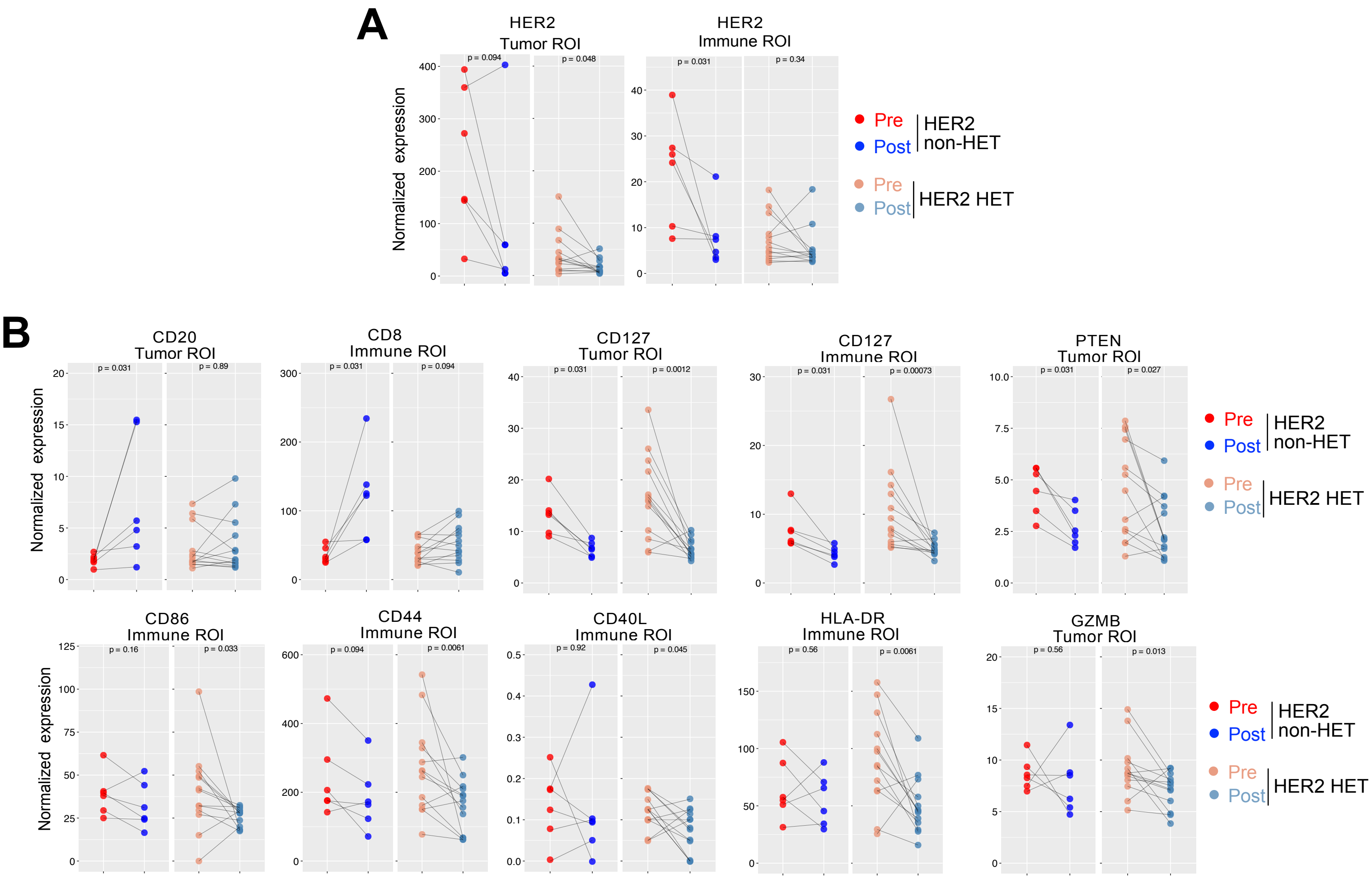
A
HER2 Tumor ROI
p = 0.094
p = 0.048
HER2 Immune ROI
p = 0.031
p = 0.34
Normalized expression
Pre
Post
HER2 non-HET
Pre
Post
HER2 HET
B
CD20 Tumor ROI
p = 0.031
p = 0.89
CD8 Immune ROI
p = 0.031
p = 0.094
CD127 Tumor ROI
p = 0.031
p = 0.0012
CD127 Immune ROI
p = 0.031
p = 0.00073
PTEN Tumor ROI
p = 0.031
p = 0.027
CD86 Immune ROI
p = 0.16
p = 0.033
CD44 Immune ROI
p = 0.094
p = 0.0061
CD40L Immune ROI
p = 0.92
p = 0.045
HLA-DR Immune ROI
p = 0.56
p = 0.0061
GZMB Tumor ROI
p = 0.56
p = 0.013

Suppl Fig. 8

Supplemental Figure 8. Treatment-induced changes in the immune environment. (**A,B**) Line plots showing the normalized expression of HER2 (A) and other immune-related targets (B) from DSP profiling among the four subgroups based on HER2 heterogeneity and pre-/post-treatment samples from the same patient. Tumor or immune ROI are indicated. P values were calculated by two-tailed Wilcoxon matched-pairs signed rank test in all panels.

SUPPLEMENTAL TABLE LEGENDS

Supplemental Table 1. Inventory of profiled sample and metadata. List of all 285 samples for RNA-seq profiling and their associated metadata including HER2 heterogeneity identification, pCR status, RCB categories, hormone receptor status, *ERBB2* FISH amplified cell percentage and HER2 IHC score. NE stands for “not evaluated”.

Supplemental Table 2. Genes list of differential expression analysis output. Lists of differentially expressed genes from the four comparisons: non-HET/nopCR vs non-HET/pCR (pre-treatment samples), HET/nopCR vs non-HET/pCR (pre-treatment samples), Pretreatment vs Posttreatment samples (non-HET/nopCR) and Pretreatment vs Posttreatment samples (HET/nopCR). Log2Fold change and adjusted p values are shown to each individual gene.

Supplemental Table 3. HER2 immunohistochemistry score and heterogeneity shift. List of 50 patients with their associated HER2 heterogeneity calling and HER2 immunohistochemistry score in pre-treatment biopsies and residual tumors. Patients showing shift of HER2 heterogeneity are indicated.

Supplemental Table 4. Inventory of *PIK3CA* and *ERBB2* hotspot variants. List of samples harboring *PIK3CA* or *ERBB2* hotspot variants called from RNA-seq. Allele frequencies are listed for each variant.

**Sample**	**Patient**	**Variant gene**	***PI3KCA* exon21**	***PIK3CA* exon9**	***ERBB2* exon23**
101_site_1_core_2	101	*PIK3CA*	H1047R (48%)		
101_site_2_core_1	101	*PIK3CA*	H1047R (14%)		
109_site_1_core_2	109	*PIK3CA*		Q546K (20%)	
115_site_1_core_1	115	*PIK3CA*	H1047R (50%)		
115_site_2_core_2	115	*PIK3CA*	H1047R (69%)		
116_site_1_core_2	116	*PIK3CA*	H1047R (23%)		
120_site_1_core_1	120	*PIK3CA*		Q546R (31%)	
120_site_2_core_1	120	*PIK3CA*		Q546R (23%)	
123_Residual_Disease	123	*PIK3CA*	H1047R (29%)		
123_site_1_core_1	123	*PIK3CA*	H1047R (38%)		
123_site_2_core_1	123	*PIK3CA*	H1047R (51%)		
124_site_2_core_1	124	*PIK3CA*	H1047R (91%)		
137_site_2_core_2	137	*PIK3CA*		E545K (55%)	
143_Residual_Disease	143	*PIK3CA*	H1047R (25%)		
161_site_2_core_2	161	*PIK3CA*	H1047R (33%)		
27_site_2_core_1	27	*PIK3CA*	H1047R (73%)		
32_Residual_Disease	32	*PIK3CA*	H1047L (33%)		
43_site_1_core_2	43	*PIK3CA*	H1047R (51%)		
46_Residual_Disease	46	*PIK3CA*	H1047R (33%)		
48_site_1_core_3	48	*ERBB2*			V777M (25%)
68_site_1_core_2	68	*PIK3CA*	H1047R (40%)		
72_site_1_core_2	72	*PIK3CA*		E542K (35%)	
74_Residual_Disease	74	*ERBB2*			V777L (85%)
74_site_1_core_2	74	*ERBB2*			V777L (38%)
74_site_2_core_2	74	*ERBB2*			V777L (84%)
83_site_2_core_1	83	*ERBB2*			V777L (73%)
91_site_2_core_2	91	*PIK3CA*		E545K (21%)	
93_Residual_Disease	93	*ERBB2*			V777L (64%)
93_site_1_core_1	93	*ERBB2*			V777L (65%)

Supplemental Table 5. Summary of key findings. List of eight biomarker/features investigated in this study with their correlative summary including 1) association with pCR and if it is different in HET and non-HET groups; 2) difference in nopCR to pCR groups split by HER2 heterogeneity, and 3) T-DM1/P induced changes split by HER2 heterogeneity.

Feature	Method	Relationship to pCR	Association with HER2 HET	Difference in no-pCR vs. pCR patients pre-treatment		T-DM1/P treatment-induced differences	
				HER2 non-HET	HER2 HET	HER2 non-HET	HER2 HET
**HER2 heterogeneity**	FISH	Only present in no pCR patients	Yes	No difference	Fewer *ERBB2* amplified cells	Decreased by treatment	No significant difference
***ERBB2* mRNA expression**	RNA-seq	Lower in no pCR patients	Yes	Lower than in pCR patients	Lower than in pCR and non-HET/nopCR patients	Increased by treatment	Same trend as HER2 non-HET tumors but no statistic difference
**ERBB2 signaling signature**	RNA-seq	Lower in HER2 non-HET/no pCR patients	Yes	Lower than in pCR patients	No significant difference	Increased by treatment	Increased by treatment
**PAM50 subtype**	RNA-seq	Higher basal and lower HER2 in no pCR patients	Yes	Higher basal and lower HER2 than in pCR patients	Higher basal and lower HER2 than in pCR patients	Decreased basal and HER2 and increased LumA features	Same trend as HER2 non-HET tumors but no statistic difference
**Treatment-induced transcriptomic response**	RNA-seq	Only analyzed in no pCR patients	Yes	NA	NA	Strong transcriptomic response	Weak transcriptomic response
**Stromal Score**	RNA-seq	Lower in HER2 HET/no pCR patients	Yes	No significant difference	Lower than in pCR patients	Increased by treatment	No significant difference
**Immune Score**	RNA-seq	Lower in HER2 non-HET/no pCR patients	Yes	Lower than in pCR patients	No significant difference	Increased by treatment	No significant difference
**BCR/TCR richness and diversity**	RNA-seq	Lower in HER2 non-HET/no pCR patients	Yes	Lower than in pCR patients	No significant difference	Increased by treatment	No significant difference
